# Novel *Miscanthus* genotypes selected for different drought tolerance phenotypes show enhanced tolerance across combinations of salinity and drought treatments

**DOI:** 10.1093/aob/mcz009

**Published:** 2019-03-10

**Authors:** Evangelia Stavridou, Richard J Webster, Paul R H Robson

**Affiliations:** 1 Institute of Biological, Environmental and Rural Sciences, Aberystwyth University, Aberystwyth, UK; 2 Institute of Applied Biosciences, Centre for Research and Technology Hellas, Thessaloniki, Greece; 3 School of Natural Sciences and Psychology, Liverpool John Moores University, Liverpool, UK

**Keywords:** Bioenergy, *Miscanthus*, photosynthesis, plant physiology, salinity tolerance, drought tolerance, abiotic stress, lignin, ash, proline, C_4_ crop

## Abstract

**Background and Aims:**

Water deficit and salinity stresses are often experienced by plants concurrently; however, knowledge is limited about the effects of combined salinity and water deficit stress in plants, and especially in C4 bioenergy crops. Here we aim to understand how diverse drought tolerance traits may deliver tolerance to combinations of drought and salinity in C4 crops, and identify key traits that influence the productivity and biomass composition of novel *Miscanthus* genotypes under such conditions.

**Methods:**

Novel genotypes used included *M. sinensis* and *M. floridulus* species, pre-screened for different drought responses, plus the commercial accession *Miscanthus × giganteus* (*M×g.*). Plants were grown under control treatments, single stress or combinations of water deficit and moderate salinity stress. Morphophysiological responses, including growth, yield, gas exchange and leaf water relations and contents of proline, soluble sugars, ash and lignin were tested for significant genotypic and treatment effects.

**Key Results:**

The results indicated that plants subjected to combined stresses showed more severe responses compared with single stresses. All novel drought-tolerant genotypes and *M×g*. were tolerant to moderate salinity stress. Biomass production in *M. sinensis* genotypes was more resilient to co-occurring stresses than that in *M×g.* and *M. floridulus*, which, despite the yield penalty produced more biomass overall. A stay-green *M. sinensis* genotype adopted a conservative growth strategy with few significant treatment effects. Proline biosynthesis was species-specific and was triggered by salinity and co-occurring stress treatments, mainly in *M. floridulus*. The ash content was compartmentalized differently in leaves and stems in the novel genotypes, indicating different mechanisms of ion accumulation.

**Conclusions:**

This study highlights the potential to select novel drought-tolerant *Miscanthus* genotypes that are resilient to combinations of stress and is expected to contribute to a deeper fundamental knowledge of different mechanistic responses identified for further exploitation in developing resilient *Miscanthus* crops.

## INTRODUCTION

The area of arid and saline land is increasing and is expected to have a major impact on future crop productivity. The environmental stresses resulting from climate change and unsustainable irrigation practices are predicted to impact crop productivity and reduce the area of available land for agriculture by 2–9 % globally and by 11–17 % within Europe (Zhang and Cai, 2011). Land not suitable for food production may be ideal for growing dedicated bioenergy crops (Oliver *et al.*, 2009), as this would reduce competition for higher-grade land and maintain lower-grade land in cultivation. Second-generation perennial biomass crops that are tolerant to environmental stress conditions could contribute to the reduction in greenhouse gas emissions through sustainable production of biomass for energy and biorenewable products, while limiting competition with food crops (Somerville *et al.*, 2010; Cai *et al.*, 2011; Popp *et al.*, 2014). Since irrigation of biomass crops is unlikely to be economic, it is important to identify genotypes that optimize the use of water in different climatic regions and those that are tolerant to salinity and water-deficit stresses.

In nature, plants are usually subjected to combinations of stresses, such as water deficit and salinity, concurrently throughout the growing season (Suzuki *et al.*, 2014; Pandey *et al.*, 2017). However, the mode of interaction between salinity and water deficit is largely unknown and the literature on combined effects of abiotic stresses in C4 bioenergy crops, especially water deficit and salinity, is limited. The combination of different stresses is experienced by plants as a new state of abiotic stress that entails a unique acclimation response or even a conflicting or antagonistic response, rather than the sum of the two stresses (Mittler, 2006; Suzuki *et al.*, 2014). Physiological responses to drought include control of stomatal aperture, decreased photosynthetic activity, altered cell-wall elasticity and the generation of toxic metabolites causing plant cell death (Ahuja *et al.* 2010), whereas under prolonged salinity, in addition to water deficits, plants are subjected to further stress due to the toxicity of the accumulated ions in the plant (Amini *et al.*, 2007; Chaves *et al.*, 2009). Under long-term water deficit, photosynthesis declines due to reduced stomatal conductance and CO_2_ uptake alongside increased photoinhibition (Pinheiro and Chaves 2011). Stomatal behaviour under water deficit may vary as different species adopt different signalling mechanisms to initiate stomatal closure (Wilkinson and Davies, 2010). A severe water deficit may provoke stomatal limitation, but in decreasing photosynthetic potential the metabolic limitations become more important (Ghannoum *et al.*, 2003; Ripley *et al.*, 2007). The metabolic limitations in C4 NADP-ME Panicoid species have been showed to be greater in relation to C3 panicoid species when exposed to severe water deficit (Ripley *et al.*, 2010).

Plant growth requires precise control of cell division and irreversible cell-wall expansion to enlarge cells in response to water uptake (Tardieu *et al.*, 2011; Feng *et al.*, 2016). Osmotic-associated stresses like salinity and water deficit interfere with plant water balance and cause reduction in cell turgor, which affects the ability of the cell to expand (Schopfer, 2006; Shao *et al.*, 2008; Benešová *et al.*, 2012) prior to any inhibition of photosynthesis or respiration (Cramer *et al.*, 1994; Cramer, 2003; Hummel *et al.*, 2010). Growth is considered the most sensitive physiological process in response to water deficit, limited by the plant’s ability to conduct osmotic adjustment (Cramer *et al.*, 2011), because the differential between the cytoplasm and the extracellular environment plays a key role in determining the direction of water movement (Feng *et al.*, 2016). The extent of cell wall deformation depends on the severity of water loss and rigidity of the cell wall affecting the biosynthesis of new cell-wall components, such as cellulose, hemicellulose and pectin (Le Gall *et al.*, 2015; Wang *et al.*, 2016) and modifies enzymatically the rheological properties of the cell wall, through the activity of reactive oxygen species on cell-wall enzymes (Skirycz *et al.*, 2010).

Resistance mechanisms against abiotic stresses can be categorized as mechanisms of avoidance and tolerance. Avoidance responses to water deficit involve changes mainly in plant anatomy or physiology for escaping stress and include increased biomass allocation to roots, leaf shedding, leaf rolling and low stomatal conductance (Touchette *et al.*, 2009).

Salinity initially occurs as an osmotic stress and gradually evolves into ionic toxicity stress. Salt stress reduces the rate of photosynthesis due to stomatal limitation or non-stomatal effects. These include decreased chlorophyll content and leaf senescence related to the accumulated ions (Johnston *et al.*, 1984; Lacerda *et al.*, 2003) and alterations in leaf photochemistry and carbon metabolism (Chaves *et al.*, 2009). Salt avoidance is defined as the selective exclusion of toxic salt absorption from the root system, the excretion of salts from the salt glands of specific halophytes and the effective partitioning of the salts in vacuoles to avoid the toxic effect during cell metabolic processes. Salts inhibit plant growth and affect metabolism and physiological processes by decreasing net photosynthesis in higher plants (Maxwell and Johnson, 2000; Baker, 2008) and impact negatively on crop productivity. Salinity has two main components affecting plant growth. Initially the water potential is lowered, and the plant experiences an osmotic stress similar to water deficit, associated with concentrated solutes in the root zone. The subsequent ionic imbalance as salts perturb the uptake of nutrients and the accumulation of ions over time is the main cause of toxicity (Munns *et al.*, 1995; Flowers, 2004; Verslues *et al.*, 2006; Munns and Tester, 2008). Many different traits contribute to salinity tolerance, which are species- and developmental stage-dependent (Munns, 2002; Jones *et al.*, 2015). In glycophytes, salt tolerance also involves the accumulation of compatible solutes, such as proline, in the cytosol and organelles for osmotic adjustment and osmoprotection (Yokoi *et al.*, 2002; Zaki and Yokoi, 2016).

*Miscanthus* has good potential for use on underutilized or abandoned marginal land where excessive salinity and low moisture levels limit plant growth. The effect of water deficit on *Miscanthus* morphology and physiology has been previously described in *M. × giganteus* by Ings *et al.* (2013), demonstrating that elongation inhibition was the most sensitive response to water deficit. *Miscanthus × giganteus* was previously found to be tolerant to moderate salinity stress and photosynthesis was rather resilient, yet it was susceptible to high salinity concentrations >10.65 dS m^−1^ (Stavridou *et al.*, 2016). Studies have shown that *Miscanthus* can grow in coastal areas where salt spray affects plant growth (Ogura and Yura, 2008; Scheiber *et al.*, 2008; Hung *et al.*, 2009). Płażek *et al*. (2014) showed that salt levels in excess of 100 mm reduced *M. × giganteus* productivity, while *M. sinensis* accessions exhibited greater variability for salt tolerance (Sun *et al.*, 2014).

There is a need for research on the performance of biomass plants such as *Miscanthus* in response to multiple abiotic stress factors. The different phases of salinity stress, i.e. the osmotic and ionic effects, in combination with the osmotic effects of water deficit stress may induce or inhibit responses observed during the occurrence of a single stress. Here we aim to (1) understand whether selecting for drought tolerance may deliver tolerance to combinations of stress, specifically drought and salinity; (2) develop knowledge of the interactions between multiple stresses in C4 crops and identify key traits that influence the productivity of *Miscanthus* genotypes under combinations of stress; (3) evaluate salinity tolerance in selected *Miscanthus* genotypes in comparison with the commercial *M. × giganteus;* and (4) determine the impact of abiotic resistance on biomass composition. Understanding the *Miscanthus* crop responses to co-occurring salinity and water deficit stresses may have significant, practical and ecological impacts on the improvement of abiotic stress tolerance of C4 crops and facilitate breeding towards genetic improvement of renewable crops for biomass and biofuel production on underutilized or abandoned land.

## MATERIALS AND METHODS

### Plant material and experimental design

The experiment was performed in a controlled glasshouse environment with a photoperiod of 16 h of daylight from supplementary lighting, with an average of 500 μmol photons m^2^ s^−1^ of photosynthetically active radiation (PAR), and 8 h of night. The temperature was set at a day/night cycle of 25/15 °C. *Miscanthus* genotypes [*Miscanthus × giganteus*, *Miscanthus sinensis* 1 (*M. sin.* 1), *Miscanthus sinensis* 2 (*M. sin.* 2) and *Miscanthus floridulus*] were selected for this study based on their previous performance under water-deficit stress (E. Stavridou, R. Webster and P. Robson, IBERS, Aberystwyth, UK, unpubl. res.). Rhizome pieces with an approximate weight of 20 g were grown in 6.2-L pots containing John Innes No. 2 compost. When plants of each genotype reached approximately the fourth or fifth leaf, they were selected from the larger group to produce an experimental population with the smallest possible variance. The selected plants of each genotype were then split into four groups with similar means and standard deviations (day 0). Water deficit and/or salinity treatments were applied at roughly the time of emergence of the fifth to seventh leaf of the main (longest) stem and all single-leaf measurements were performed on the youngest leaf with a fully expanded ligule on that stem. Treatments were applied relative to the field capacity (FC) of the soil as described below. The four treatments were: 80 % FC (control, C), 15 % FC (water deficit, D), 60 mm NaCl at 80 % FC (salinity, S) and the combination of 15 % FC and 60 mm NaCl (water deficit and salinity, S+D) with six replicates per treatment. Salinity stress, induced by applying 60 mm NaCl, was chosen on the basis of a previous study of *M. × giganteus* grown in a range of salt concentrations (Stavridou *et al.*, 2016; E. Stavridou, IBERS, Aberystwyth, UK, unpubl. res.), this level of salinity was moderate and induced an osmotic rather than ionic stress in *M. × giganteus*. A water stress FC of 15 % was chosen from previous studies that showed that 20 % FC was very mild and did not significantly impact growth of various *Miscanthus* genotypes and 15 % FC was moderate and affected biomass and physiological responses across many diverse *Miscanthus* genotypes (Malinowska *et al.*, 2017; M. Malinowska and P. Robson, IBERS, Aberystwyth, UK, unpubl. res.). The experimental set-up was a randomized split-plot design with four treatments and four genotypes per block. Blank pots, with soil only, were used to determine water evaporation from the soil.

Water was applied gravimetrically and target weights were adjusted for the biomass of each genotype using regular harvests of the above-ground biomass from plants growing in the same conditions. The amount of water added to the 15 and 80 % FC treatments was calculated from the difference in weights. The target weight of the pot (P_T_) was calculated as follows:

$${\text{P}}_{T}={\text{P}}_{D}+{\text{M}}_{W}+\text{FC}\times \left({\text{P}}_{W}-{\text{P}}_{D}\right)$$ (1)

where P_D_ and P_W_ are the dry and wet weight of pots plus soil, respectively, measured prior to transplantation. To adjust for accumulated biomass through the experiment, total wet above ground biomass (M_W_) was estimated for each genotype using separate plants grown and harvested for this purpose.

The combined stress treatment was applied using 60 mm NaCl solution and the corresponding target weight for 15 % FC. Pot target weight was maintained by regular weighing and re-watering approximately every 3 d throughout the 8 weeks of the experiment.

### Morphological measurements

All morphological measurements were completed once a week. The length of the longest stem was measured from the base of the stem at soil level to the fully expanded ligule of the youngest leaf. Leaf area was assessed by measuring the length and width (at half leaf length) of the youngest fully expanded leaf with a ligule and was calculated as described by Clifton-Brown and Lewandowski (2000):

$${\text{L}}_{A}=0.\text{74}\times {\text{L}}_{L}\times {\text{L}}_{W}$$ (2)

where L_A_ is leaf area (cm^2^), L_L_ is leaf length (cm) and L_W_ is leaf width at half L_L_ (cm). Harvested plants were separated into leaves, stems, rhizomes and roots and the final morphological parameters were measured. Plant dry matter (M_D_) was obtained after drying at 60 °C until a constant weight was achieved.

### Physiological measurements and water relations

Soil moisture content and electrical conductivity (ECp) were measured using a multi-parameter WET sensor (WET-2, Delta-T Devices Ltd, Cambridge, UK) as an average of three measurements per pot, the sensor being inserted at three roughly equidistant points around the surface of the pot. Readings were recorded by a hand-held meter (HH2, Delta-T Devices Ltd, Cambridge, UK).

The hydration state of the leaf was estimated from the leaf relative water content (L_RWC_), which is the water content of the leaf relative to its fully hydrated or fully turgid state, using the following equation:

$${\text{L}}_{\text{RWC}}=\left({\text{L}}_{F}-{\text{L}}_{D}\right)/\left({\text{L}}_{T}-{\text{L}}_{D}\right)$$ (3)

Five leaf discs from each plant were excised and placed in tubes, which were capped immediately and stored on ice until all samples were collected and weighed (L_F_). Turgid weight (L_T_) was measured from rehydrated freshly weighed leaves floating in distilled water for 3–4 h in the dark. The samples were placed in an oven at 60 °C until constant weight was measured (L_D_).

Plant water use efficiency (WUE), defined as grams of dry biomass produced per kilogram of water (Richards, 1992; Morison *et al.*, 2008), was calculated at final harvest as the ratio of total dry above-ground biomass (M_AG_, g) to total transpired water. Transpired water was the amount of water applied (W_A_) during the experiment minus the water loss from evaporation (W_E_). Gravimetric data for pots without plants were used for all treatments to adjust for evaporation of water from the surface of the soil to calculate WUE as follows:

$$\text{WUE}={\text{M}}_{\text{AG}}/\left({\text{W}}_{A}-{\text{W}}_{E}\right)$$ (4)

Intrinsic leaf water use efficiency was calculated from gas exchange of CO_2_ and H_2_O as the ratio of CO_2_ assimilation (*A*) to stomatal conductance (*g*_s_) at photon fluxes of 300 (net irradiance) and 1500 μmol m^−2^ s^−1^ (saturating irradiance), using a portable infra-red gas analyser (GFS-3000FL, Heinz Walz GmbH, Effeltrich, Germany).

The water potential (*Ψ*_leaf_) (Scholander *et al.*, 1966) of the second youngest leaf with a fully expanded ligule was measured using a pressure chamber (Skye Instruments Ltd., Llandrindod Wells, UK). Leaf samples were excised at pre-dawn and midday, placed in aluminium folders to prevent transpiration and transferred to the laboratory, where they were measured immediately after each sampling under low light conditions.

Stomatal conductance (mmol m^−2^ s^−1^) was measured using an AP4 porometer (Delta-T Devices Ltd., Cambridge, UK) according to the manufacturer’s instructions.

Relative chlorophyll content was measured according to Stavridou *et al.* (2016) on three leaves per plant using a SPAD-502 meter (Konica Minolta Optics Inc., Osaka, Japan).

Dark-adapted chlorophyll *a* fluorescence measurements were made on the youngest leaf with a fully expanded ligule on the adaxial leaf surface using a Handy PEA chlorophyll fluorimeter with dark adaptation leaf clips (Hansatech Instruments Ltd., King's Lynn, UK) after 30 min of dark adaptation, as described in Stavridou *et al.* (2016). Maximum quantum yield of PSII (*F*_v_/*F*_m_ or TR_0_/ABS, where TR_0_ is trapping of excitation energy and ABS is light absorption) was determined as follows: *F*_v_/*F*_m_ = (*F*_m_ − *F*_0_)/*F*_m_. The performance index (PI) was derived according to the Nernst equation. It is the equation that describes the forces of redox reactions and generally movements of Gibbs free energy in biochemical systems and is used to characterize plant vitality as the overall photosynthetic performance under different stresses (Strasser *et al*., 2000, 2004). The PI was calculated as: PI_ABS_ = (RC/ABS) × (TR_0_/DI_0_) × [ET_0_/(TR_0_ − ET_0_)], taking into account all the main photochemical processes, such as (1) ABS and TR_0_, (2) conversion of excitation energy to photosynthetic electron transport (ET_0_) and (3) dissipation of excess light energy absorbed by PSII (DI_0_).

### Gas exchange

Gas exchange measurements were made *in vivo* using an integrated open gas exchange system and a modulated chlorophyll fluorimeter (GFS-3000FL, Heinz Walz GmbH, Effeltrich, Germany). The responses of assimilation (*A*) to photosynthetic photon flux density (*Q*) (*A*/*Q*) and to intercellular CO_2_ concentration (*A*/*C*_i_ curves) were obtained from the first leaf with a fully expanded ligule. All gas exchange and fluorescence results were graphed on an absorbed light basis. Measurements were made at 2, 4 and 8 weeks.

Leaf absorptance (*α*) was measured from 400 to 700 nm, by inserting the leaf in an assembly with defined aperture between two aligned integrating spheres (SpectroClip-JAZ-TR, Ocean Optics, Oxford, UK) following the protocol of Webster *et al.* (2016). The average transmittance (*τ*) and reflectance (*R*) (*n* = 5) were used to determine *α* = (1 − *R* − *τ*) along the area of leaf that had been within the gas exchange cuvette. This was used to calculate the total light absorbed (*Q*_abs_) by the leaves as described in Webster *et al.* (2016) and Naidu and Long (2004), with a proportion of 90/10 % red/blue light. The maximum quantum yield corrected for absorbed irradiance was calculated from the initial slope of the light response curve (Webster *et al.*, 2016):

$$\Phi_{(\text{CO2},}{}_{\text{max})}=\Phi_{\text{max}}/\alpha$$ (5)

### Photosynthetic intercellular CO_2_ response curves

The response of net leaf CO_2_ uptake (*A*) to intracellular CO_2_ concentration (*C*_i_) was assessed on three leaves. Leaves were placed in the leaf cuvette. Air temperature was controlled at 25 °C and the vapour pressure deficit (VPD) was maintained between 1 and 2 kPa with a *C*_i_ of 400 μmol mol^−1^. Leaves were dark-adapted for 30 min and a measurement of *F*_v_/*F*_m_ was recorded. Leaves were illuminated with 1500 μmol m^−2^ s^−1^ and allowed to acclimate for ~10 min (Long and Bernacchi, 2003) or until a stable *A* had been achieved, then a measurement was recorded. Concentrations were then changed stepwise to the following *C*_i_ values in sequence: 400, 1000, 1500, 2000, 750, 400, 200, 150, 100 and 50 μmol mol^−1^. The leaf remained at each concentration until a stable *A* could be determined. An empirical non-rectangular hyperbola was used to describe the dependence of *A* on *C*_i_, and to predict the variables of the C4 model (von Caemmerer, 2000) the Excel fitting tool (Bellasio *et al.*, 2016) was used.

### Photosynthetic light response curves

Measurements of *A* versus *Q* were performed in parallel with *A*/*C*_i_ curves in the GFS-3000F integrated modulated chlorophyll PAM fluorοmeter. Leaves were placed in the leaf cuvette of the GFS-3000FL and light-adapted to an incident photon flux of 1500 μmol m^−2^ s^−1^; prior to measurements the CO_2_ was set to 400 μmol mol^−1^. Photosynthetic and light-adapted fluorescence parameters were measured at the following actinic light levels in sequence: 400, 1000, 1500, 2000, 750, 400, 300, 200, 150, 100 and 50 μmol m^−2^ s^−1^. At each light level, once a new steady state was reached gas exchange rates were recorded. Simultaneously, to estimate changes in the quantum yield of non-cyclic electron transport, *Φ*PSII = *F*_m_′ − *F*_s_/*F*_m_′ was determined (Genty *et al.*, 1989). The rate of dark respiration, *R*_dark_ [mmol (CO_2_) m^−2^ s^−1^], was calculated from the light response curve fit based on the model by Ye (2007).

### Biochemical responses

Proline was extracted using a cold extraction procedure according to Carillo *et al.* (2008) by mixing 20 mg of leaf fresh weight aliquots with 400 mL of ethanol:water (40:60 v/v). Proline content was measured spectrophotometrically using the method of Carillo *et al.* (2011) from three biological and three technical replicates per treatment. The ash content (%) was determined as previously described in Stavridou *et al.* (2016).

Lipid peroxidation was estimated based on the protocol by the total content of 2-thiobarbituric acid-reactive substances (TBARS) expressed as equivalents of malondialdehyde (MDA), a decomposition product of polyunsaturated fatty acids that has been utilized as a biomarker for lipid peroxidation (Mittler, 2002), as described in Stavridou *et al.* (2016). The amount of MDA2-thiobarbituric acid complex (red pigment) was calculated on a fresh weight (W_F_) basis from the excitation coefficient, ε = 155 mm cm^−1^ according to eqn (6) and soluble sugars according to eqn (7) (Li *et al.* 2008):

$$\text{MDA }\left(\;\text{​}\mu \text{​}{\text{ mol g}}^{-\text{1}}{\text{W}}_{F}\right)=\{[\text{6}.\text{45}\times (A_{\text{532}}-A_{\text{6}00})\;0.\text{56}\times A_{\text{45}0}]/\text{1}000\}\;(\;\text{​}\mu \text{​}{\text{ mol mL}}^{-\text{l}})\times V\left(\text{mL}\right)/{\text{W}}_{F}\left(\text{g}\right)\;$$ (6)

$$\text{soluble sugar content }\left({\text{mmol g}}^{-\text{1}}{\text{W}}_{F}\right)=\left(\text{11}.\text{74}\times A_{\text{45}0}/\text{1}000\right)\;\left({\text{mmol mL}}^{-\text{1}}\right)\times V\left(\text{mL}\right)/{\text{W}}_{F}\left(\text{g}\right)$$ (7)

where *A*_450_, *A*_532_ and *A*_600_ are the absorbances at 450, 532 and 600 nm, respectively, and *V* is the volume of the extract solution.

For cell-wall isolation, 70 mg of air-dried finely ground plant biomass was weighed in 2-mL screw-cap tubes and processed according to Foster *et al.* (2010) with some modifications, as follows: digestion was performed using 0.01 % sodium azide (NaN_3_), amylase (50 μg mL^−1^ H_2_O; from *Bacillus* sp.; Sigma) and pullulanase (18.7 units from *Bacillus acidopullulyticus*; Sigma).

For the acetyl bromide determination of lignin, all samples were assayed in triplicate with three technical replicates each and a standard sample also in triplicate at intervals to correct for baseline drift, following the procedure described by Foster *et al.* (2010) and da Costa *et al.* (2014). From each sample 200 μL was transferred to a UV-transparent 96-well plate (UV-Star; Greiner Bio-One, UK) and the absorbance at 280 nm of each assay mixture was measured three times with a microplate reader (μQuant; Bio-Tek Instruments, Winooski, VT, USA) using KC4 software (v. 3.3; Bio-Tek).

An assay control sample of a standard cell wall preparation was included in all batches of the lignin assay as an internal standard. Additionally, negative controls containing no cell wall material were included and their absorbance at 280 nm was set as the absorbance baseline. A specific absorption coefficient (SAC) of 17.78 g^−1^ L cm^−1^ has been reported for purified HCl-dioxane lignin from *Miscanthus* samples (Lygin *et al.*, 2011) and this was used to calculate the percentages of lignin in the cell wall biomass samples on a dry weight basis using the following equation:

$$\text{ABSL }\left(\%\right)=A_{\text{28}0}\times \text{SAC}\times {\text{P}}_{L}\times {\text{V}}_{R}\times {\text{W}}_{S}\times \text{1}00\%$$ (8)

where ABSL is the percentage of acetyl bromide-soluble lignin, *A*_280_ is the absorption reading at 280 nm, P_L_ is the path length determined for the 96-well microplates with a volume of 200 μL per well used during the analysis (0.556 cm), V_R_ is the reaction volume (L) and W_S_ is the sample weight (g).

### Statistical analyses

Statistical analyses were performed using R (R Core Team, 2016). The effects of genotype, treatment and time (d) on the morphological and physiological parameters were assessed using three-way repeated measures ANOVA (for genotypes, treatments, days and their interactions), and the final morphological parameters at harvest and the biochemical parameters were assessed using two-way-ANOVA (for genotypes, treatments and their interactions) using the afex package (Singmann *et al.*, 2016). All data were tested for normality (Shapiro test) and, if normality failed and homogeneity passed, transformations were attempted. For the three-way ANOVA, data were also tested with Mauchly’s test for sphericity and if the assumption of sphericity was violated the corresponding Greenhouse–Geisser corrections were performed. If significant differences were found among treatments, Tukey’s HSD *post hoc* test was performed to determine specific treatment differences using the agricolae package (de Mendiburu 2016).

## RESULTS

### Biomass

There was a genotypic difference in accumulated above-ground biomass when plants were grown in well-watered (C) treatments, with the highest levels in *M. × giganteus* and *M. floridulus* genotypes; *M. sin*. 1 accumulated intermediate levels and *M. sin.* 2 the lowest. Genotypic differences in biomass were reduced after stress treatments. The water deficit treatment (D) had the least effect, resulting in a moderate reduction in biomass accumulation relative to C in *M. × giganteus* and *M. floridulus*, but not in either *M. sin*. 1 or *M. sin.* 2. Salinity (S)-containing treatments resulted in a significant reduction in biomass in all four genotypes relative to C treatment. Above-ground biomass accumulated to lower levels in S and S+D treatments compared with C and D treatments in all genotypes, but these reductions were not significant in the *M. sinensis* genotypes. The genotypic differences in accumulated biomass disappeared when plants were grown in S+D treatments as all plants accumulated similar levels of biomass (Table 1). Combining data across all four treatments, genotypes *M. × giganteus* and *M. floridulus* accumulated greater total biomass than *M. sin*. 1 and *M. sin.* 2 and the total accumulated biomass declined in the order C > D > S > S+D (Table 1).

**Table 1.** Main effects of genotype and treatment on accumulated dry biomass: above-ground dry matter (AG M_D_), leaf dry matter (L_D_), stem dry matter (S_D_), below-ground dry matter (BG M_D_), rhizome dry matter (RZ M_D_), root dry matter (R_D_) and total dry matter (Total M_D_)

Main effects	AG M_D_	T_HSD_	L_D_	T_HSD_	S_D_	T_HSD_	BG M_D_	T_HSD_	RZ M_D_	T_HSD_	R_D_	T_HSD_	Total M_D_	T_HSD_
Genotype														
* M. × giganteus*	34.7 ± 2.0	ab	10.9 ± 0.63	b	23.8 ± 1.4	a	63.7 ± 2.6	a	45.9 ± 2.1	a	17.8 ± 0.8	b	98.5 ± 4.3	a
* M. floridulus*	40.3 ± 2.4	a	13.8 ± 0.85	a	26.5 ± 1.6	a	71.9 ± 4.9	a	45.2 ± 2.1	a	26.7 ± 3.4	a	112.3 ± 7.1	a
* M. sin*. 1	31.9 ± 1.5	b	13.3 ± 0.6	ab	18.6 ± 0.9	b	34.0 ± 2.2	b	25.9 ± 1.8	b	8.1 ± 0.6	c	66.0 ± 3.4	b
* M. sin.* 2	24.7 ± 1.3	c	13.4 ± 0.8	ab	11.3 ± 0.6	c	37.9 ± 2.9	b	19.2 ± 1.2	b	18.7 ± 1.9	b	62.5 ± 4.1	b
Treatment														
* *C	38.9 ± 2.4	a	15.0 ± 0.71	a	23.9 ± 2.0	a	58.8 ± 5.9	ab	38.6 ± 3.4	a	20.2 ± 3.6	ab	97.8 ± 8.2	a
* *D	35.6 ± 2.1	ab	14.0 ± 0.79	a	21.5 ± 1.6	ab	59.5 ± 4.6	a	36.5 ± 3.1	a	23.1 ± 2.4	a	95.1 ± 6.2	a
* *S	29.6 ± 1.9	bc	11.3 ± 0.71	b	18.3 ± 1.4	ab	47.0 ± 4.1	ab	33.2 ± 3.1	a	13.8 ± 1.3	b	76.6 ± 5.7	ab
* *S+D	27.6 ± 1.3	c	11.1 ± 0.52	b	16.6 ± 1.2	b	42.2 ± 2.7	b	27.9 ± 2.1	a	14.3 ± 1.1	b	69.7 ± 3.6	b

Data are mean ± s.e. (*n* = 6).

T_HSD_, Tukey HSD *post hoc* test. Different lowercase letters indicate significance at *P* < 0.05.

The proportions of leaf and stem in above-ground biomass differed between genotypes. *Miscanthus floridulus*, *M. sin*. 1 and *M. sin.* 2 produced significantly higher dry biomass as leaf compared with *M. × giganteus*; stem dry biomass was significantly higher in *M. floridulus* and *M. × giganteus* than in the two *M. sinensis* genotypes. The general trends combining all four genotypes was that leaf biomass decreased significantly in treatments involving salinity (S and S+D), whilst stem *biomass* decreased only under S+D. In *M. × giganteus* both leaf and stem biomass declined in S and S+D treatments, whereas in *M. floridulus* reduction in leaf biomass in S+D was the main effect on above-ground M_D_. Both leaf and stem biomass decreased in the two *M. sinensis* genotypes in treatments involving salinity (S and S+D), but this was not significant (Tables 1 and 2 and Supplementary Data Table S1).

**Table 2.** Interaction effect between genotype and treatment on accumulated dry biomass: above-ground dry matter (AG M_D_), leaf dry matter (L_D_), stem dry matter (S_D_), below-ground M_D_ (BG M_D_), rhizome M_D_ (RZ M_D_), root dry matter (R_D_) and total dry matter (Total M_D_)

Genotype	Treatment	AG M_D_	T_HSD_	L_D_	T_HSD_	S_D_	T_HSD_	BG M_D_	T_HSD_	RZ M_D_	T_HSD_	R_D_	T_HSD_	Total M_D_	T_HSD_
*M. × giganteus*	C	44.78 ± 2.55	a	14.12 ± 0.9	a	30.6 ± 2.69	a	70.2 ± 6.14	a	52.1 ± 5.22	a	18.2 ± 1.41	ab	115.1 ± 8.3	a
	D	36.25 ± 4.23	ab	11.3 ± 1.34	ab	24.9 ± 2.99	ab	68.4 ± 4.41	a	46.4 ± 3.39	a	22.1 ± 1.46	a	104.7 ± 8.3	a
	S	28.67 ± 3.37	b	8.71 ± 1.03	b	19.9 ± 2.4	b	59.7 ± 6.56	a	45 ± 5.17	a	14.72 ± 1.5	b	88.4 ± 9.2	a
	S+D	29.25 ± 1.97	b	9.54 ± 0.31	b	19.7 ± 1.8	b	56.5 ± 2.34	a	40.1 ± 2.47	a	16.4 ± 0.97	b	85.8 ± 2.8	a
*M. floridulus*	C	48.87 ± 5.62	a	17.0 ± 1.88	a	31.8 ± 3.8	a	86.8 ± 15	a	50.7 ± 3.7	a	36.9 ± 12.1	a	135.7 ± 20.4	a
	D	42.21 ± 3.52	ab	14.2 ± 1.18	ab	28 ± 2.55	a	82.4 ± 6.37	ab	49.9 ± 3.72	a	31.7 ± 4.13	a	124.6 ± 8.6	ab
	S	39.51 ± 4	ab	14.1 ± 1.37	ab	25.4 ± 2.69	a	67.85 ± 4.3	ab	47.1 ± 2.79	a	20.7 ± 1.88	a	107.36 ± 7.8	ab
	S+D	30.81 ± 4	b	9.85 ± 1.05	b	20.9 ± 2.97	a	50.6 ± 2.9	b	33.04 ± 2.2	b	17.5 ± 1.22	a	81.42 ± 6.87	b
*M. sin*. 1	C	35.8 ± 2.29	a	14.8 ± 1.06	a	21 ± 1.39	a	40.7 ± 5.94	a	32.3 ± 5.53	a	8.37 ± 0.63	ab	76.51 ± 6.7	a
	D	36.26 ± 4.4	a	15.2 ± 1.78	a	21 ± 2.63	a	38.0 ± 4.82	a	26.5 ± 3.57	a	11.4 ± 2.01	a	74.3 ± 8.6	a
	S	28.75 ± 1.7	a	11.5 ± 0.61	a	17.2 ± 1.36	a	29.9 ± 2.07	a	24.0 ± 1.95	a	5.94 ± 0.44	b	58.71 ± 3.4	a
	S+D	27.1 ± 1.56	a	11.76 ± 0.8	a	15.3 ± 0.9	a	27.3 ± 2.21	a	20.8 ± 1.83	a	6.55 ± 0.45	b	54.5 ± 3.64	a
*M. sin.* 2	C	26.3 ± 2.43	a	14.21 ± 1.6	a	12.1 ± 0.96	a	37.6 ± 3.75	a	20.1 ± 1.93	a	17.5 ± 1.97	a	63.93 ± 5.9	a
	D	27.6 ± 3.28	a	15.3 ± 1.78	a	12.3 ± 1.5	a	49.2 ± 9.23	a	22.2 ± 3.72	a	27 ± 5.92	a	76.85 ± 12	a
	S	21.5 ± 2.88	a	11.03 ± 1.7	a	10.5 ± 1.35	a	30.5 ± 3.47	a	16.5 ± 1.27	a	13.9 ± 2.38	a	51.97 ± 6.3	a
	S+D	23.51 ± 1.99	a	13.1 ± 1.14	a	10.3 ± 1.03	a	34.4 ± 2.78	a	17.8 ± 1.34	a	16.5 ± 2.1	a	57.14 ± 4.5	a

Data are mean ± s.e. (*n* = 6).

T_HSD_, Tukey HSD *post hoc* test. Different lowercase letters indicate significance at *P* < 0.05.

Across all treatments *M. × giganteus* and *M. floridulus* produced almost twice as much below-ground biomass as did the two *M. sinensis* genotypes and below-ground biomass was significantly lower in the S+D treatment. Below-ground biomass was lower in genotypes growing in treatments involving S but the variance in these measurements was particularly high and therefore these effects were mostly not significant (Tables 1 and 2 and Supplementary Data Table S2). Only in the *M. floridulus* genotype after S+D treatment was there a significant decrease in below-ground biomass, and this was mainly due to a significant reduction in rhizome. No other significant effects of treatment on rhizome dry biomass were detected. The *M. floridulus* genotype produced significantly higher root biomass across all treatments, *M. × giganteus* and *M. sin.* 2 produced similar levels of root biomass, and *M. sin.* 1 produced significantly lower root biomass than the other three genotypes across all treatments. Root *dry matter (RD)* produced by *M. × giganteus* and *M. sin*. 1 was significantly reduced after S and S+D treatments and increased after D compared with C plants. Levels of root biomass produced by *M. floridulus* and *M. sin.* 2 were not significantly affected by any treatment (Tables 1 and 2 and Supplementary Data Table S2). Treatment did not affect the ratio of below-/above- ground M_D_ (B/A M_D_), but there was a genotypic effect, with the B/A M_D_*ratio decreasing in the order M. × giganteus* and *M. floridulus* > *M. sin.* 2 > *M. sin*. 1.

### Growth parameters

**Fig. 1.**
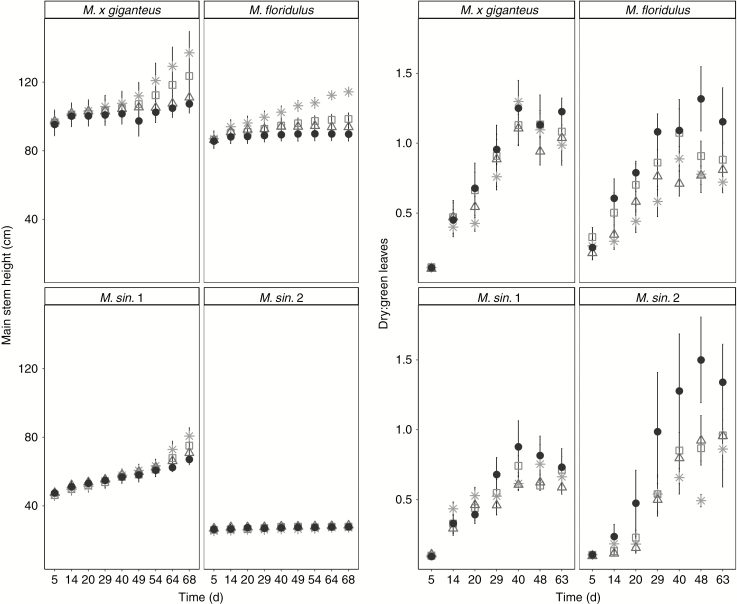
Height (cm) (left panels) and dry:green leaf ratio (right panels) of the main stem over time for *M. × giganteus*, *M. sin*. 1, *M. sin.* 2 and *M. floridulus* in response to control (asterisks), drought (squares), salinity (triangles) and salinity plus drought (black circles) treatments over the 67-d experimental period. Data are mean ± s.e. (*n* = 6).

*Miscanthus × giganteus* were the tallest plants, followed by *M. floridulus*, *M. sin*. 1 and *M. sin.* 2 (Fig. 1). Under control conditions, height increased over time in all genotypes except *M. sin.* 2, and under stress conditions stem elongation declined over time in all genotypes except *M. sin*. 1, which continued to grow under all stress conditions (Supplementary Data Table S3). The relative reduction in final height under stress conditions was significant only for genotype *M. floridulus*. Stem length in the different treatments did not differ significantly between consecutive days in any genotype, except in *M. floridulus* from day 49 onwards under S+D, and from day 64 onwards in D and S treatments (Fig. 1).

*When all treatments were combined, the M. × giganteus* and *M. floridulus* and *M. sin.* 1 genotypes produced a higher leaf number compared with *M. sin.* 2. Over time, leaf number increased in all treatments except in the combined S+D, where leaf number was maintained at a constant level. At harvest the different stress treatments resulted in reduced leaf number compared with the control treatment, plants in S produced significantly fewer leaves, and leaf numbers were moderately lower in plants growing in D and S+D treatments (Table 3). The dry:green leaf ratio was significantly affected by the stresses and genotypes in a cumulative way (Fig. 1). The S+D combined stress significantly increased the proportion of dry leaves in *M. sin.* 2 and *M. floridulus* compared with C and S conditions. Under S+D, *M. sin.* 1 had the lowest proportion of dry leaves and *M. floridulus* the highest number of dry leaves. In C and S treatments both *M. sinensis* genotypes had the lowest dry:green leaf ratio compared with the other two genotypes.

**Table 3.** Main effects of genotype and treatment on growth parameters at harvest. Average values and Tukey HSD (T_HSD_) *post hoc* test for the main effects of genotype and treatment on morphological data

Main effects	Height (cm)	T_HSD_	Leaf number on main stem	T_HSD_	Total leaf number	T_HSD_	Leaf area (cm^2^)	T_HSD_
Genotype								
* M. × giganteus*	119.7 ± 4.54	a	14.6 ± 0.34	b	40.12 ± 1.47	ab	60.4 ± 9.169	bc
* M. floridulus*	99.08 ± 2.58	b	19.8 ± 1.36	a	46.8 ± 2.38	a	96.02 ± 4.83	a
* M. sin.* 1	73.4 ± 2.36	c	13.75 ± 0.3	b	39.12 ± 1.65	ab	42.18 ± 3.31	c
* M. sin.* 2	27.87 ± 0.7	d	10.58 ± 0.26	c	34.16 ± 1.85	b	79.6 ± 4.34	ab
Treatment								
* *C	45.0 ± 9.19	a	15.5±1.26	a	44.21±1.9	a	55.08±6.68	a
* *D	38.1 ± 7.78	a	15.79±1.05	a	41.21±2.21	ab	78.4±7.35	a
* *S	32.7 ± 6.68	a	13.79±0.62	a	37.91±2.48	b	69.59±7.07	a
* *S+D	31.4 ± 6.41	a	13.75±0.9	a	36.91±1.18	ab	75.2±6.75	a

Data are mean ± s.e. (*n* = 6).

T_HSD_, Tukey HSD *post hoc* test. Different lowercase letters indicate significance at *P* < 0.05.

At harvest the number of stems (Tables 3 and 4 and Supplementary Data Table S3) was relatively unchanged over time in S+D stress, whereas under C, D and S conditions stem number increased. The number of stems was unaffected in *M. × giganteus*, *M. sin.* 2 and *M. floridulus* growing under control or stress conditions, with an increase in stem number at harvest. The number of stems in *M. sin*. 1 at harvest was greatly affected by S+D and moderately affected by S and D treatments.

**Table 4.** Interaction effect between genotype and treatment on the growth parameters at harvest

Genotype	Treatment	Height (cm)	T_HSD_	Leaf number on main stem	T_HSD_	Total leaf number	T_HSD_	Leaf area (cm^2^)	T_HSD_	Stem number	T_HSD_
*M. × giganteus*	C	137 ± 12.54	a	15 ± 0.36	a	47.3 ± 3.08	a	17.5 ± 7.96	b	5.3± 1.108	a
	D	123.5 ± 8.38	a	14.16 ± 0.91	a	36 ± 5.41	a	87.3 ± 18.85	a	4.83± 0.31	a
	S	111 ± 3.808	a	15.16 ± 0.31	a	36.8 ± 2.65	a	58.18 ± 18.75	ab	4.16± 1.33	a
	S+D	107.3 ± 5.38	a	14.3 ± 0.95	a	40.3 ± 1.11	a	78.5 ± 14.05	ab	5.5± 0.91	a
*M. floridulus*	C	114.3 ± 2.67	a	23.16 ± 3.32	a	51.5 ± 4.78	a	82.26 ± 6.76	a	5.5± 0.7	a
	D	98.5 ± 3.48	b	23.16 ± 1.79	a	47.33 ± 4.91	a	101.36 ± 8.16	a	5.6± 1.28	a
	S	93.8 ± 4.24	b	15.66 ± 1.76	a	50.16 ± 5.19	a	91.8 ± 8.61	a	5.83± 0.84	a
	S+D	89.6 ± 4.02	b	17.3 ± 2.78	a	38.33 ± 3.04	a	108.2 ± 12.86	a	5± 1.15	a
*M. sin.* 1	C	80.6 ± 4.95	a	13.3 ± 0.49	a	41.16 ± 2.33	a	37.5 ± 5.63	a	8.16± 0.94	a
	D	75.08 ± 6	a	14.3 ± 0.88	a	43.16 ± 4.57	a	38.12 ± 5.43	a	6.5± 0.428	ab
	S	70.9 ± 3.48	a	13.8 ± 0.6	a	37.33 ± 3.49	a	39.4 ± 7.86	a	6.16± 0.6	ab
	S+D	67.08 ± 3.19	a	13.5 ± 0.43	a	34.83 ± 1.81	a	53.6 ± 6.62	a	5.16± 0.4	b
*M. sin.* 2	C	27.3 ± 1.53	a	10.5 ± 0.43	a	36.83 ± 1.85	ab	82.9 ± 5.95	a	5.6± 0.49	a
	D	27.8 ± 1.47	a	11.5 ± 0.43	a	38.33 ± 0.66	a	86.8 ± 9.14	a	6± 0.51	a
	S	28.08 ± 1.63	a	10.5 ± 0.56	a	27.33 ± 3.87	b	88.9 ± 7.25	a	4± 0.68	a
	S+D	28.25 ± 1.36	a	9.83 ± 0.6	a	34.16 ± 2.62	ab	59.9 ± 8.22	a	6± 0.577	a

Data are mean ± s.e. (*n* = 6).

T_HSD_, Tukey HSD *post hoc* test. Different lowercase letters indicate significance at *P* < 0.05.

Stem diameter was not affected by the stress treatments and showed only significant genotypic effects (*P* < 0.05), in the order *M. × giganteus* > *M. sinensis* > *M. floridulus*. The number of nodes decreased in the order *M. × giganteus* > *M. floridulus* > *M. sin.* 1 > *M. sin.* 2 (data not shown).

The area of a standard leaf (the youngest leaf with a ligule) increased over time in all genotypes, but under stress the increase was statistically significant only in *M. × giganteus (*Tables 3 and 4 and Supplementary Data Table S3*).* A significant decrease in the area of the standard leaf was recorded only in genotype *M. sin.* 2 under severe S+D stress from day 29 onwards.

### WUE and water relations

There was a significant genotype and treatment effect associated with WUE for above-ground M_D_. WUE was maintained under S and S+D stresses in both *M. sinensis* genotypes and *M. × giganteus*. WUE increased significantly under the stress treatments only in *M. floridulus* (Supplementary Data Fig. S1). Genotype *M. sin.* 2 produced biomass having the lowest WUE. The values ranged from 4.7 to 5.2 g M_D_ kg^−1^ H_2_O for *M. × giganteus* and from 3.7 to 6.2 g M_D_ kg^−1^ H_2_O for *M. floridulus* under S+D combined stress. The WUE of biomass produced by *M. sin.* 1 ranged from 5.8 to 8.2 g kg^−1^ H_2_O in salinity stress. The WUE of accumulated total M_D_ (above- and below-ground biomass) increased under all stresses, moderately under D and more intensely under S and S+D combined stress. Under S+D, mean values for WUE reached 15.5 g kg^−1^ H_2_O for *M. × giganteus* and 16.5 g kg^−1^ H_2_O for *M. floridulus*, whilst under salinity the WUE of *M. sin.* 1 reached 16.6 g kg^−1^ H_2_O.

Transpiration was affected by genotype and treatment. Transpiration rate was relatively stable in *M. sin.* 2 under all stresses, whereas transpiration in the other three genotypes was reduced in S and was most significantly reduced by S+D. Transpiration in *M. floridulus* was also significantly lower in the D treatment compared with C (Supplementary Data Fig. S1).

**Fig. 2.**
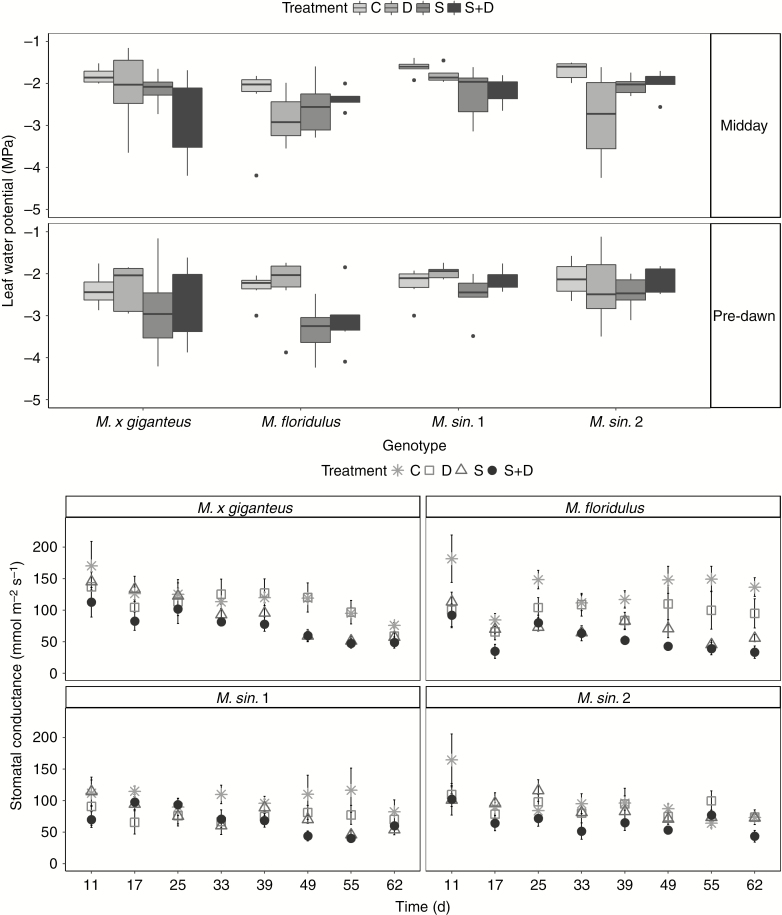
Leaf water potential [median (second quartile; horizontal line) and first and third quartiles (dots indicate outliers)] and stomatal conductance (mean ± s.e.) for *M. × giganteus*, *M. sin.* 1, *M. sin.* 2 and *M. floridulus* in response to control (C), drought (D), salinity (S) and salinity and drought (S+D) treatments over the 67-d experimental period (*n* = 6).

Stomatal conductance (Fig. 2) was significantly affected by treatments and the transitory effect of time (d). Under S and D, *g*_s_ was significantly lower than in non-stressed plants, and a further dramatic decline was observed under S+D, indicating that plants under severe stress severely restrict stomata. There was significant variation in *g*_s_ for each genotype in response to time (Supplementary Data Table S4). The effect of treatment over time indicated that under C and S involved stresses *g*_s_ declined over time, the decline was not significant in the D treatment. All genotypes reduced *g*_s_ significantly under combined S+D stress. Stomatal conductance of *M. floridulus* and *M. sin.* 1 was also reduced under D and S and that of *M. × giganteus* under S, but stomatal conductance in *M. sin.* 2 was only responsive to S+D.

Leaf water potential (Fig. 2) was significantly affected by treatment, genotype and time point (pre-dawn and midday) and an interaction between treatment and time of day was observed. At pre-dawn no statistical differences were observed between plants in stress treatments and well-watered plants in any genotype (Fig. 2). The two *M. sinensis* genotypes had similar *Ψ*_leaf_, *M. floridulus* had the lowest *Ψ*_leaf_ (highest negative pressure) and *M. × giganteus* did not differ significantly from the rest. Overall, and regardless of the treatments and genotypes, *Ψ*_leaf_ had higher negative pressure at midday compared with pre-dawn (Supplementary Data Table S5). Under stress treatments involving S (S and S+D), *Ψ*_leaf_ was significantly more negative compared with control plants, whereas D had a moderate effect on *Ψ*_leaf_. At pre-dawn, only under S was *Ψ*_leaf_ significantly lower compared with C, whereas at midday plants under D and S+D combined stress reduced their *Ψ*_leaf_ dramatically. The value of *Ψ*_leaf_ was stable between pre-dawn and midday under D and S+D combined stress, whereas an increase (lower negative pressure) was observed under C and S conditions during midday (Fig. 2, Supplementary Data Table S5).

The value of L_RWC_ was not significantly affected by treatment, but there was a significant genotypic effect. It was significantly higher in *M. sin.* 2 compared with *M. floridulus*, in which L_RWC_ was lowest. Genotypes *M. sin.* 1 and *M. × giganteus* had moderate levels of L_RWC_ and no significant differences from *M. sin.* 2 and *M. floridulus* (Supplementary Data Fig. S3).

Soil moisture (m^3^ m^−3^) changed significantly in response to the main effects of genotype, treatment and days and the interaction effects between genotype and days and between treatment and days (*P* < 0.001). Soil moisture was significantly higher in the S treatment (a, *P* < 0.05), followed by S+D and C treatments (b, *P* < 0.05), and plants grown under the D treatment had the lowest soil moisture content (c, *P* < 0.01) (Supplementary Data Fig. S4). Different letters indicate significant differences between treatments. Nevertheless, plants growing under C and S treatments were consistently maintained at 80 % FC and D and S+D at 15 % FC (Supplementary Data Fig. S5).

### Maximum quantum efficiency of PSII and relative chlorophyll content

**Fig. 3.**
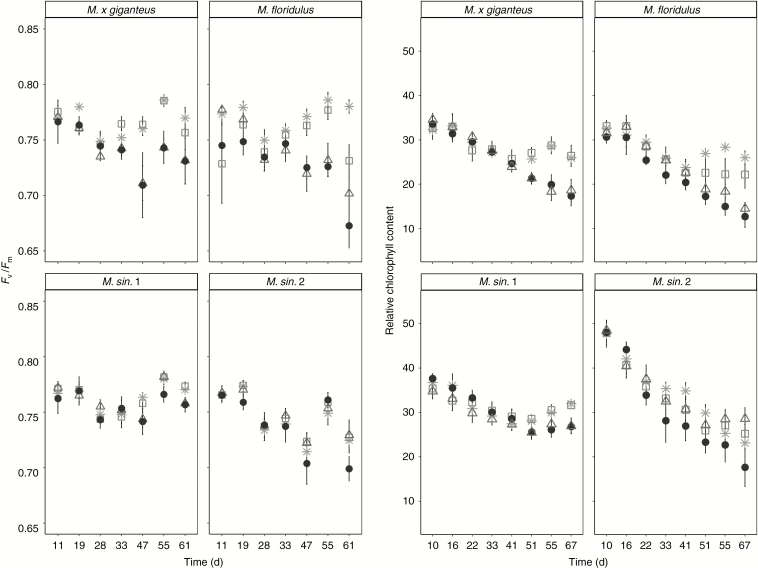
Dark-adapted photosystem II maximum quantum efficiency (*F*_v_/*F*_m_) (left panels) and relative chlorophyll content (right panels) of *M. × giganteus*, *M. sin.* 1, *M. sin.* 2 and *M. floridulus* in response to control (asterisks), drought (squares), salinity (triangles) and salinity plus drought (black circles) treatments over the 67-d experimental period. Data are mean ± s.e. (*n* = 6).

Dark-adapted PSII maximum quantum efficiency (*F*_v_/*F*_m_) (Fig. 3, Table 5 and Supplementary Data Table S6) was significantly reduced under S and the S+D combined stress condition, whereas D did not affect *F*_v_/*F*_m_ compared with non-stressed plants. Genotypes *M. × giganteus* and *M. floridulus* showed significant declines in *F*_v_/*F*_m_ when grown under S and S+D conditions, whereas the maximum quantum efficiency of *M. sinensis* genotypes was maintained under all stress conditions (Table 5). The PI was significantly reduced after day 28 (Supplementary Data Table S7). Genotype *M. floridulus* had significantly lower PI compared with the other genotypes (Table 6). The PI decreased moderately in D stressed plants and was further reduced under S and significantly declined in S+D stress. The *M. sinensis* genotypes were both more tolerant to S and S+D stresses than *M. × giganteus* and *M. floridulus (*Table 6*).*

**Table 5.** Main effects of genotype and treatments on maximum quantum efficiency (*F*_v_/*F*_m_) of *Miscanthus* genotypes. Tukey HSD (T_HSD_) *post hoc* test for interaction effect between genotype and treatment for *F*_v_/*F*_m_

Treatment	*M. × giganteus*		*M. sin*. 1		*M. sin.* 2		*M. floridulus*	
	*F*_v_/*F*_m_	T_HSD_	*F*_v_/*F*_m_	T_HSD_	*F*_v_/*F*_m_	T_HSD_	*F*_v_/*F*_m_	T_HSD_
C	0.766 ± 0.003	a	0.764 ± 0.003	a	0.742 ± 0.004	a	0.771 ± 0.003	a
D	0.764 ± 0.003	a	0.763 ± 0.003	a	0.747 ± 0.004	a	0.751 ± 0.006	ab
S	0.742 ± 0.005	b	0.76 ± 0.003	a	0.746 ± 0.004	a	0.738 ± 0.006	bc
S+D	0.743 ± 0.006	b	0.756 ± 0.003	a	0.737 ± 0.005	a	0.728 ± 0.006	c

Data are mean ± s.e. (*n* = 42).

Different lowercase letters indicate significant differences between treatments for each genotype (*P* < 0.05).

**Table 6.** Interaction effect between treatment on performance index (PI) of PSII. Average value and Tukey HSD (T_HSD_) *post hoc* test for interaction effect between genotype and treatment

Treatment	Genotype	PI	T_HSD_	Genotype	Treatment	PI	T_HSD_
C	*M.* × *giganteus*	1.4 ± 0.075	a	*M.* × *giganteus*	C	1.4 ± 0.075	ab
	*M. floridulus*	1.38 ± 0.068	a		D	1.44 ± 0.073	a
	*M. sin.* 1	1.45 ± 0.072	a		S	1.16 ± 0.073	b
	*M. sin.* 2	1.53 ± 0.11	a		S+D	1.15 ± 0.082	b
D	*M.* × *giganteus*	1.44 ± 0.07	a	*M. floridulus*	C	1.38 ± 0.068	a
	*M. floridulus*	1.14 ± 0.09	b		D	1.14 ± 0.09	ab
	*M. sin*. 1	1.44 ± 0.06	a		S	1.05 ± 0.073	b
	*M. sin.* 2	1.49 ± 0.13	ab		S+D	0.77 ± 0.057	c
S	*M.* × *giganteus*	1.16 ± 0.073	b	*M. sin. 1*	C	1.45 ± 0.072	a
	*M. floridulus*	1.05 ± 0.073	b		D	1.44 ± 0.069	a
	*M. sin*. 1	1.27 ± 0.06	ab		S	1.27 ± 0.067	a
	*M. sin.* 2	1.54 ± 0.11	a		S+D	1.23 ± 0.073	a
S+D	*M.* × *giganteus*	1.15±0.082	a	*M. sin.* 2	C	1.53 ± 0.118	a
	*M. floridulus*	0.77 ± 0.057	b		D	1.49 ± 0.118	a
	*M. sin*. 1	1.23 ± 0.073	a		S	1.54 ± 0.112	a
	*M. sin*. 2	1.33 ± 0.103	a		S+D	1.33 ± 0.10	a

Data are mean ± s.e. (*n* = 42).

Different lowercase letters indicate significant differences between genotypes for each treatment and between treatments for each genotype (*P* < 0.05).

Relative chlorophyll content (Fig. 3) was reduced significantly under stress treatments, particularly under S and S+D combined stress, when compared with the C treatment, while D induced a moderate reduction. Overall, the leaves of genotype *M. sin.* 2 had a higher relative chlorophyll content compared with *M. sin.* 1, *M. × giganteus* and *M. floridulus*. Salinity and combined stress had the most significant effect by reducing relative chlorophyll content of *M. × giganteus* and *M. floridulus* and, later in the experiment, *M. sin.* 1. Genotype *M. sin.* 2 did not show any significant differences in relative chlorophyll content between treatments but levels were lowest in the combined treatment towards the end of the experiment.

### Leaf light absorptance and CO_2_ assimilation rate

Light absorptance (*Q*_abs_) was unaffected in all genotypes by water deficit. However, *Q*_abs_ was decreased in treatments S and severely reduced in the combined S+D treatment. The S+D combined stress had a cumulative effect, reducing *Q*_abs_ sequentially over the duration of the study.

The assimilation rate in ambient (*A*, 400 PAR) and saturating light (*A*_sat_, 2000 PAR) and in response to saturating intracellular CO_2_ (*A*_max_) was significantly reduced under S and S+D combined stress over time, indicating damage induced by the accumulated ions in the plants. Values of *A*_sat_ and *A* were greatest in *M. × giganteus*, followed by *M. sin.* 1, *M. floridulus* and *M. sin.* 2, which had the lowest assimilation rate (Table 7). *A*_sat_ was unaffected under D and was moderately reduced (by 24.7 %) under S and significantly reduced (by 33.0 %) under severe S+D stress compared with non-stressed plants. In ambient light, *A* was reduced significantly under all stresses, with the greatest reduction observed in the S+D treatment. The assimilation rate of *M. sin.* 2 was not affected by the stress treatments in response to either light or intracellular CO_2_ (Table 5). The *A*_sat_ of *M. sin.* 1 was maintained under all treatments (Table 7). The *A*_max_ was reduced slightly, but not significantly, under D and S treatments, but was greatly reduced in S+D stress compared with the C plants. The S and S+D stresses significantly reduced *A*_max_ in *M. × giganteus* and *M. floridulus.* Assimilation rate was not affected by D or S in either *M. sinensis* genotype. Also, *M. × giganteus*, regardless of treatment effect, had the highest assimilation rate of the four genotypes (Table 6).

**Table 7.** Effect of genotype and treatment on photosynthesis. Average value and Tukey HSD (T_HSD_) *post hoc* test for interaction effect between genotype and treatment for assimilation rates in saturating light (*A*_SAT_) and ambient light (*A*), in response to saturating intracellular CO_2_ (*A*_max_, μmol m^−2^ s^−1^)_,_ stomatal conductance (*g*_s,_ mmol m^−2^ s^−1^) and light-saturated gross assimilation rate (GA_sat_)

Genotype	Treat-ment	*A*_SAT_	T_HSD_	*A*	HSD	*A*_max_	T_HSD_	*g*_s_	HSD	GA_sat_	T_HSD_
*M. × giganteus*	C	13.7 ± 0.56	ab	9.2 ± 0.42	ab	13.5 ± 0.74	ab	59.3 ± 3.78	ab	14.2 ± 0.61	ab
	D	15.2 ± 0.81	a	10.2 ± 0.59	a	16.1 ± 0.76	a	63.4 ± 4.64	a	15.5 ± 0.99	a
	S	12.5 ± 1.03	ab	8.3 ± 0.64	ab	11.5 ± 0.81	bc	49.2 ± 3.70	ab	12.4 ± 1.07	ab
	S+D	10.9 ± 1.20	b	7.4 ± 0.89	b	9.8 ± 1.06	c	45.6 ± 5.76	b	11.0 ± 1.15	b
*M. floridulus*	C	14.6 ± 0.90	a	9.9 ± 0.67	a	13.2 ± 1.08	a	68.9 ± 5.41	a	16.5 ± 1.79	a
	D	11.4 ± 1.45	ab	7.7 ± 0.93	ab	10.6 ± 1.33	ab	43.6 ± 5.52	b	11.5 ± 1.59	b
	S	9.2 ± 0.58	b	5.6 ± 0.33	b	7.7 ± 0.49	bc	33.7 ± 1.39	b	9.10 ± 0.48	b
	S+D	8.2 ± 0.77	b	5.3 ± 0.56	b	6.8 ± 0.73	c	30.5 ± 2.31	b	8.16 ± 0.81	b
*M. sin.* 1	C	11.3 ± 0.66	a	7.8 ± 0.47	a	9.7 ± 0.79	a	50.2 ± 3.76	a	11.2 ± 0.85	a
	D	10.8 ± 0.71	a	7.4 ± 0.46	ab	8.4 ± 0.78	a	43.9 ± 3.57	ab	10.3 ± 0.86	a
	S	9.8 ± 0.61	a	6.9 ± 0.44	ab	6.8 ± 0.85	ab	38.1 ± 2.57	ab	9.5 ± 0.71	a
	S+D	9.2 ± 0.68	a	5.6 ± 0.54	b	5.1 ± 0.57	b	32.8 ± 3.12	b	8.57 ± 0.63	a
*M. sin*. 2	C	7.2 ± 0.60	a	4.8 ± 0.38	a	5.9 ± 0.54	a	33.3 ± 3.29	a	6.7 ± 0.56	a
	D	8.5 ± 0.68	a	5.4 ± 0.64	a	6.6 ± 0.77	a	36.5 ± 3.58	a	7.39 ± 0.84	a
	S	7.6 ± 0.73	a	5.1 ± 0.52	a	5.9 ± 0.53	a	33.4 ± 4.18	a	7.14 ± 0.67	a
	S+D	8.4 ± 0.70	a	5.3 ± 0.62	a	6.6 ± 0.93	a	34.8 ± 4.49	a	7.86 ± 0.66	a

Data are mean ± s.e. (*n* = 4).

Different lowercase letters in T_HSD_ columns indicate significant differences between treatments for each genotype (*P* < 0.05).

The ratio of intercellular to external CO_2_ concentration (*C*_i_/*C*_a_) was significantly reduced under S and D and was moderately reduced under S+D. Genotypes showed variation in *C*_i_/*C*_a_ under control conditions; however, this variation disappeared under the effect of stress treatments. Only genotype *M. floridulus* showed a reduction in *C*_i_/*C*_a_ between stress treatments, with the greatest decrease being observed under D conditions, whereas S and S+D treatments induced a moderate decline (Supplementary Data Fig. S2).

A 46.4 % decrease was observed in the CO_2_-saturated phosphoenol pyruvate (PEP) carboxylation rate (*V*_pmax_) in *Miscanthus* growing under S+D stress. The *V*_pmax_ decreased in *M. × giganteus* significantly under S and S+D treatments, by 55.6 and 45.6 %, respectively. A relative decrease was observed in *M. floridulus* under stress treatments, but it was significant only under S+D (65.6 %). The *V*_pmax_ in both *M. sinensis* genotypes was not affected significantly by stress, but a relative decrease was observed in *M. sin.* 1 under S (23 %) and S+D (45 %). Under C and D conditions, *M. × giganteus* had significantly higher *V*_pmax_ compared with the other genotypes, whereas under S and S+D no differences were observed between the genotypes.

Nevertheless, stomatal limitations (Ls) were not the primary cause of reduction in the assimilation rate under the stress treatments, regardless of the genotypic differences observed, i.e. *M. × giganteus* and *M. sin.* 2 had greater Ls compared with *M. sin.* 1 and *M. floridulus*, which showed significantly lower Ls. In *M. floridulus* stomatal limitation was significantly higher under S+D and moderately higher under S and D stresses. This may be attributed to the intense stomatal control under all stress treatments (Table 6).

### Efficiency of electron transport into CO_2_ fixation

**Fig. 4.**
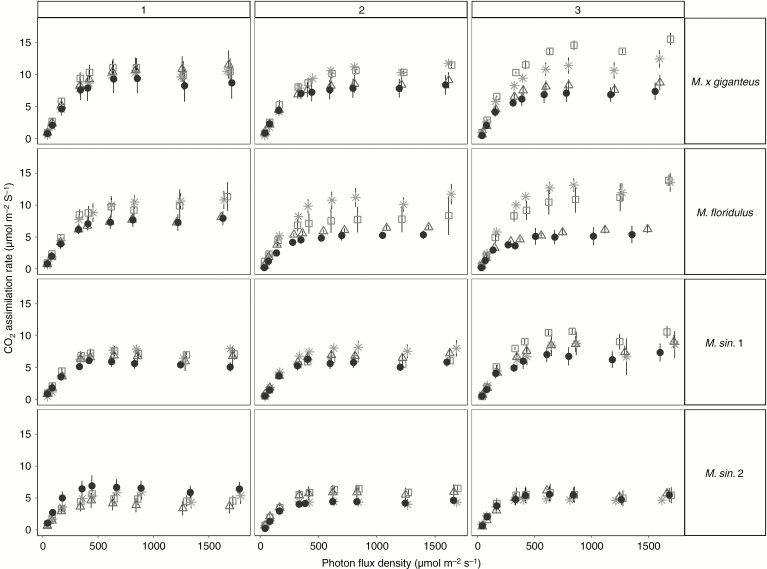
Changes in CO_2_ assimilation rate (*A*) with photon flux density (*Q*) for *M. × giganteus*, *M. sin.* 1, *M. sin.* 2 and *M. floridulus* in response to control (asterisks), drought (squares), salinity (triangles) and salinity plus drought (black circles) treatments over three time points (weeks 2, 4 and 8). Data are mean ± s.e. (*n* = 4).

The effect of stress treatments on the relationship between assimilation rate and photon flux density (*A*/*Q* curves) is shown in Fig. 4. Analysis of the ratio of quantum efficiency of photosynthetic electron transport through photosystem II to CO_2_ assimilation (φPSII/φCO_2_) indicated a significant reduction over time in control conditions, but under stress treatments the slope of φPSII/φCO_2_ was significantly lower compared with non-stressed plants and remained unchanged over time (Table 8).

**Table 8.** Interaction effect between time and treatment on φPSII/φCO_2_ and LCP

Treatment	Time point	φPSII/φCO_2_	T_HSD_ BTP	T_HSD_ WTP	LCP	T_HSD_ BTP	T_HSD_ WTP
C	W2	18.8 ± 1.01	a	A	34.5 ± 2.63	a	A
	W4	16.5 ± 0.81	ab	A	34.8 ± 1.42	a	A
	W8	14.0 ± 0.70	b	A	29.8 ± 1.36	a	A
D	W2	14.1 ± 0.79	a	B	30.5 ± 1.75	ab	A
	W4	13.6 ± 0.87	a	A	26.7 ± 2.22	b	B
	W8	14.9 ± 0.76	a	A	33.7 ± 1.24	a	A
S	W2	15.3 ± 0.86	a	B	30.1 ± 1.60	a	A
	W4	14.7 ± 0.66	a	A	28.4 ± 1.25	a	B
	W8	15.7 ± 0.76	a	A	28.8 ± 2.34	a	A
S+D	W2	14.2 ± 0.99	a	B	28.2 ± 2.35	a	A
	W4	15.4 ± 0.78	a	A	32.3 ± 2.02	a	AB
	W8	15.3 ± 0.64	a	A	31.6 ± 1.10	a	A

Data are mean ± s.e. (*n* = 4).

T_HSD_, Tukey HSD *post hoc* test; BTP, between time points; WTP, within time points.

Different lowercase letters in the T_HSD_ BTP columns indicate significant differences between time points [weeks (W) 2, 4 and 8] for the same treatment and different uppercase letters in the T_HSD_ WTP columns indicate significant differences within time points for the different treatments (*P* < 0.05).

The light compensation point (LCP) was significantly lowered under S treatment compared with the C treated plants, while S+D and D treatments induced a moderate decline in week 4 followed by a recovery phase in week 8 (Table 8). The stresses did not significantly affect respiration in light (*R*_light_) or dark (*R*_dark_); however, a reduction was observed under D and S stresses for the former and only under S for the latter.

### Biochemical responses to treatments

**Fig. 5.**
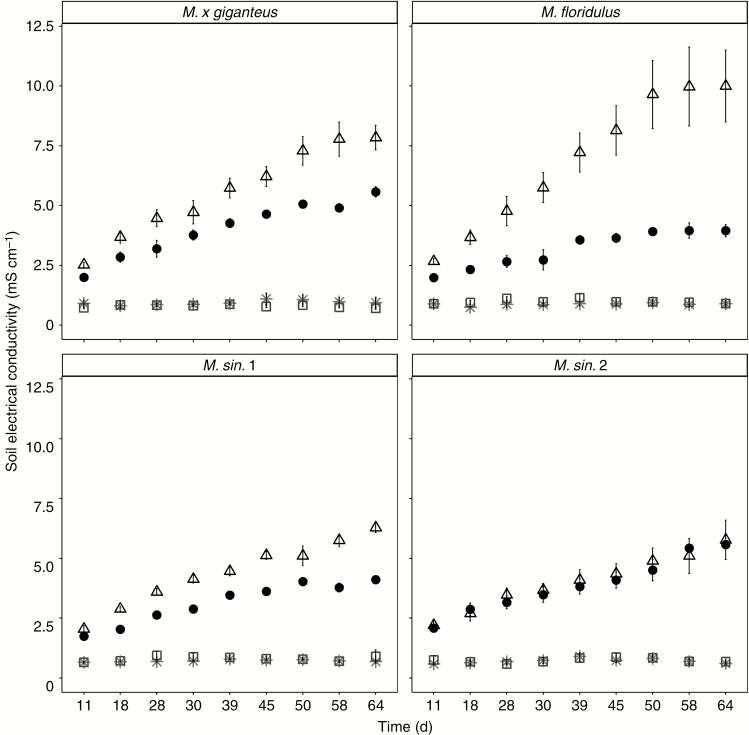
Soil electrical conductivity for *M. × giganteus*, *M. sin*. 1, *M. sin.* 2 and *M. floridulus* in response to control (asterisks), drought (squares), salinity (triangles) and salinity plus drought (black circles) treatments over the 67-d experimental period. Data are mean ± s.e. (*n* = 6).

**Fig. 6.**
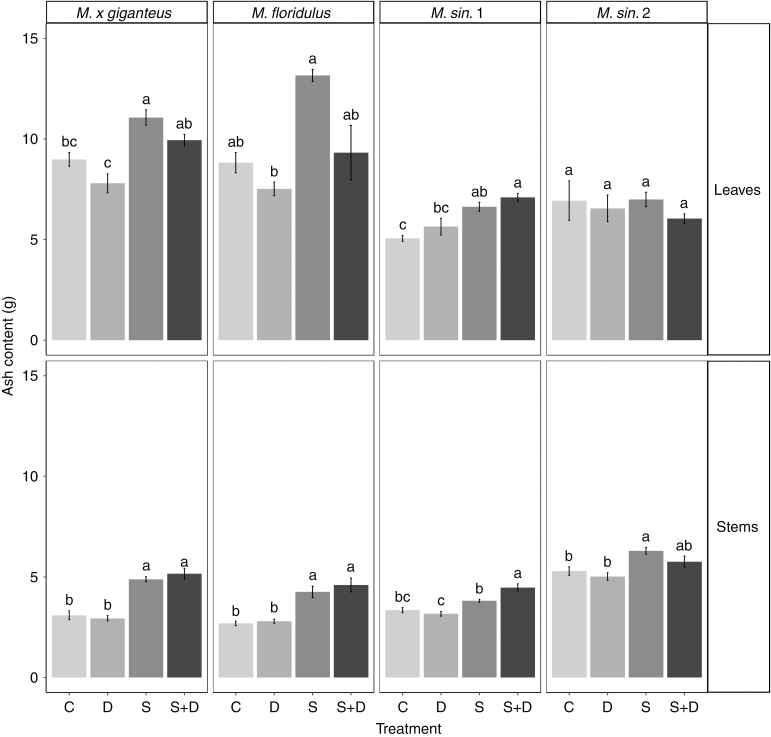
Ash content in the leaves and stems of *M. × giganteus*, *M. sin*. 1, *M. sin.* 2 and *M. floridulus* in response to control (C), drought (D), salinity (S) and salinity plus drought (S+D) treatments. Data are mean ± s.e. (*n* = 6). Different letters indicate significant differences between treatments for each genotype (*P* < 0.05).

Proline content was significantly affected by the interaction effect between genotype and treatment. Proline increased under all stress conditions in all genotypes, but a significant increase was observed only in *M. floridulus* (Table 9). The S and S+D treatments induced a dramatic increase in proline in M. floridulus (Table 9), which with respect to the S treatment, could be attributed to the higher amount of salt accumulated in the pots compared to the other genotypes (Fig. 5) or/and to the higher amount of ash observed in the leaves (Fig. 6 and Table 10).

**Table 9.** Interaction between genotype and treatment on proline content (μmol g–1 FW)

Genotype	*M. × giganteus*		*M. sin 1*		*M. sin 2*		*M. floridulus*	
Treatment	Proline	T_HSD_	Proline	T_HSD_	Proline	T_HSD_	Proline	T_HSD_
C	1.05 ± 0.23	a	2.03 ± 0.51	a	1.9 ± 0.28	a	1.38 ± 0.12	b
D	2.19 ± 0.78	a	2.26 ± 0.88	a	1.68 ± 0.33	a	1.82 ± 0.25	b
S	7.68 ± 4.67	a	1.32 ± 0.17	a	4.56 ± 2.64	a	34.1 ± 16.34	a
S+D	5.4 ± 1.89	a	1.65 ± 0.336	a	2.09 ± 0.44	a	55.8 ± 22.08	a

Data are mean ± s.e. (*n* = 6). FW, fresh weight of the sample.

T_HSD_, Tukey HSD *post hoc* test. Different lowercase letters in T_HSD_ columns indicate significant differences between treatments for each genotype (*P* < 0.05).

Malondialdehyde content was not affected by treatment or genotype. All genotypes had the lowest MDA content in the D treatment and a trend of increased MDA was observed in *M. × giganteus* and *M. sin*. 1 plants growing under S+D stress (Supplementary Data Table S8), indicating higher lipid peroxidation.

Soluble sugars were affected significantly by treatment (*P* < 0.05) and not significantly by the interaction between genotype and treatment (*P* < 0.1). Increased content of soluble sugars was observed under single S and D treatments, while C and S+D treatments showed reduced soluble sugars. Only genotype *M. sin.* 2 showed a significant increase in soluble sugars, in the orders D > S and S+D > C (Supplementary Data Table S8).

### Ash and lignin contents

*Miscanthus* ash content (Fig. 6 and Table 10) was higher under S and S+D treatments, whereas plants growing in C and D conditions had lower ash content. Leaves had significantly higher ash content compared with stems. The lowest ash content was observed in genotype *M. sin*. 1 compared with the other three genotypes. The genotypes *M. × giganteus* and *M. sin*. 1 had increased amount of leaf ash content under S and S+D treatments compared with the control plants, *M. floridulus* showed a significantly higher amount of ash in leaves only under the S treatment. However, in genotype *M. sin.* 2 the treatments did not affect ash in the leaves but rather in stems, where ash accumulated under S and S+D treatments (Fig. 6 and Table 10). Interestingly, under treatments involving a drought component (D and S+D), *M. × giganteus* and *M. floridulus* genotypes showed lower ash content compared with C and S treatments, respectively. Stem ash content was significantly higher under S and S+D combined stress compared with C and D treatments in all genotypes; however, the effects of combined S+D stress on the stem ash content of genotype *M. sin*. 1 was more severe compared with the S treatment (Fig. 6 and Table 10). All genotypes under all treatments showed lower ash content in stems compared with leaves except *M. sin.* 2 under C, S and S+D treatments, in which no significant differences were observed between the two tissue types (Fig. 6 and Table 10).

**Table 10.** Effect of treatment, genotype and type of tissue on biomass quality properties. Average values and Tukey HSD (T_HSD_) *post hoc* test for interaction effect between genotype, treatment and tissue type for ash and ABSL contents (%)

Genotype		*M. × giganteus*		*M. sin*. 1		*M. sin.* 2		*M. floridulus*	
Tissue	Treatment	Ash	HSD	Ash	T_HSD_	Ash	HSD	Ash	T_HSD_
Leaves	C	8.98 ± 0.34	bc	5.06 ± 0.14	c	6.93 ± 0.98	a	8.82 ± 0.51	b
	D	7.79 ± 0.47	c	5.64 ± 0.41	bc	6.55 ± 0.65	a	7.52 ± 0.33	b
	S	11.1 ± 0.37	a	6.63 ± 0.22	ab	6.99 ± 0.36	a	13.15 ± 0.3	a
	S+D	9.94 ± 0.29	ab	7.09 ± 0.19	a	6.04 ± 0.23	a	9.32 ± 1.35	b
Stems	C	3.09 ± 0.22	b	3.35 ± 0.13	bc	5.28 ± 0.21	b	2.69 ± 0.11	b
	D	2.94 ± 0.13	b	3.17 ± 0.11	c	5.02 ± 0.18	b	2.79 ± 0.11	b
	S	4.8 ± 0.12	a	3.81 ± 0.08	b	6.29 ± 0.16	a	4.26 ± 0.28	a
	S+D	5.15 ± 0.26	a	4.47 ± 0.18	a	5.76 ± 0.25	ab	4.59 ± 0.34	a
Tissue	Treatment	ABSL	HSD	ABSL	HSD	ABSL	HSD	ABSL	HSD
Leaves	C	11.7 ± 0.56	a	13.9 ± 0.36	a	15.6 ± 0.92	a	11.5 ± 0.44	a
	D	11.9 ± 0.49	a	14.1 ± 0.67	a	13.3 ± 0.53	a	12.3 ± 0.75	a
	S	11.2 ± 0.47	a	13.4 ± 0.43	a	15.1 ± 0.89	a	11.2 ± 0.31	a
	S+D	11.4 ± 0.56	a	13.5 ± 0.46	a	14.3 ± 0.37	a	11.8 ± 0.54	a
Stems	C	17.4 ± 0.86	ab	17.8 ± 0.33	a	18.3 ± 0.89	a	18.7 ± 0.53	a
	D	17.8 ± 1.12	ab	17.9 ± 0.39	a	20.2 ± 0.73	a	18.6 ± 0.55	a
	S	18.8 ± 0.65	a	17.3 ± 0.45	a	19.3 ± 0.54	a	19.2 ± 0.41	a
	S+D	15.5 ± 0.48	b	18.07 ± 0.24	a	18.3 ± 0.99	a	19.4 ± 0.56	a

Data are mean ± s.e. (*n* = 6).

Different lowercase letters in the T_HSD_ columns indicate significant differences between treatments for each genotype (*P* < 0.05).

The percentage of acetyl bromide-soluble lignin content (ABSL) (Table 10) was affected by genotype, with *M. × giganteus* having the lowest and *M. sin.* 2 the highest percentage. There was also a main effect of tissue type, leaves having lower lignin content compared with stems. The ABSL percentage was decreased by the S treatment in stems of genotype *M. × giganteus*. Nevertheless, no significant changes were observed in any genotype and type of tissue for genotypes *M. floridulus*, *M. sin*. 1 and *M. sin.* 2 (Table 10). In all treatments and regardless of genotype, stems always had a higher lignin content compared with leaves under control conditions, except in *M. sin.* 2, where the value was similar in leaves and stems (Table 10).

## DISCUSSION

In this study the variation in morphological, physiological and biochemical responses of four *Miscanthus* genotypes were assessed under control conditions (80 % FC) and stress conditions of moderate salinity (60 mm NaCl and ~5.44 dS m^−1^ in 80 % FC), water deficit (15 % FC) and combined salinity and water deficit (60 mm NaCl in 15 % FC). All of the genotypes exhibited severe responses under combined stress treatment compared with those experiencing a single stress. The response of most parameters was similar in the *M. sinensis* genotypes and usually different from those of the fast-growing *M. × giganteus* and *M. floridulus*. The *M. sin.* 2 genotype showed no response in most parameters measured under stress and maintained a slow-growing, compact, dark green, hard and sharp foliage that could be supported by the very low stomatal conductance even under control conditions. In contrast, *M. sin.* 1 grew faster than *M. sin.* 2 and had softer leaves that were less pigmented.

Biomass yield has been identified as the key factor in determining economic viability in biomass production for economic models of bioenergy generation (Styles *et al.*, 2008). Under water deficit, *Miscanthus* genotypes maintained *MD* by increasing leaf and stem number. Moderate salinity induced a reduction in *MD* in *M. × giganteus* that was related to reduction in leaf dry matter (L_D_) and stem dry matter (S_D_). The novel *Miscanthus* genotypes when grown in moderate salinity stress were tolerant and expressed different morphophysiological responses. Co-occurring S and D stress exacerbated the losses in *MD* for *M. × giganteus* and *M. floridulus*, but not in *M. sinensis*, and all four genotypes accumulated similar levels of biomass under combined stress treatment. It has been reported that *Miscanthus* spp. from Taiwan, such as the *M. floridulus* reported in this study, are adapted to a variety of habitats from agricultural to drought and saline, resulting in several ecotypes (Chou *et al.*, 2001). The yield potential has been characterized as moderate in *M. floridulus* and higher than in *M. sinensis* (Xu *et al.*, 2015), but the latter performs well under drought conditions (Clifton-Brown *et al.*, 2001). The decrease in L_D_ under S and S+D treatments combined with an increased proportion of dry leaves in S+D and the reduction in relative chlorophyll content in S and S+D treatments in all genotypes was possibly induced by the excess Na^+^ and Cl^− ^accumulated in the leaves, as demonstrated by the increased ash content.

Sustained leaf expansion, in relation to the reduction in L_D_, especially in the S and S+D treatments, may be an anatomical adaptation to compensate for reduced assimilation (Anyia and Herzog, 2004) by conferring higher light interception and carbon gain per unit mass invested in leaves (Lambers and Poorter, 1992). The increase in L_A_ and maintenance of L_D_ under S, but decreased L_A_ and maintenance of L_D_ under S+D combined stress in *M. sin.* 2, might indicate that the severity of S+D combined stress induced the reduction in L_A_ in order to control transpiration. Bayuelo-Jiménez *et al.* (2003) suggested that the decrease in the specific L_A_ of salt-stressed plants reflects an overloading of leaves with inorganic and organic solutes, which allows osmotic adjustment but reduces the carboxylation efficiency. Indeed, *M. sin.* 2 showed a significant increase in soluble sugars under all stress treatments.

Water use efficiency was increased in *M. × giganteus*, *M. sin.* 1 (non-significantly) and *M. floridulus* under all stresses, especially under the severe S+D combined stress. *M. sin.* 2 did not show any differences in WUE under stresses, which could possibly be attributed to the low stomatal conductance of this genotype even under control conditions. Both the *M. sinensis* genotypes maintained transpiration in response to water deficit and this was maintained in *M. sin.* 2 across stress treatments indicating that *M. sin.* 2, by having low *g*_s_ even under control conditions, may tolerate stresses via a conservative growth strategy.

Regulation of *g*_s_ for control of water loss has been identified as an early event in the response to water deficit, limiting carbon uptake and appearing to occur in response to hydraulic and chemical signals (Chaves *et al.*, 2009; Wilkinson and Davies, 2010). The different techniques used here to measure *g*_s_ show differences in the values. The porometer shows the *in situ g*_s_, whereas measurements with the gas analyser are inevitably influenced by air mixing in the leaf chamber, dark adaptation of leaves and the fluctuating light and CO_2_ environment around the leaf surface. Additionally, during performing the A/*C*i and A/Q curves, the leaves were in a functional state rather than a steady state and were not acclimated to the leaf cuvette environment. One of the primary dehydration avoidance mechanisms is reduction in transpiration via stomatal control (Flexas and Medrano, 2002; Chaves *et al.*, 2009). Here, the accumulative effect of increasing salinity and the co-occurring moderate salinity and water deficit stress exacerbated stomatal closure, whereas under single abiotic stresses in isolation all genotypes demonstrated effective stomatal control to sustain leaf gas exchange. In *M. × giganteus*, L_RWC_ remained unchanged in leaves under increased salinity (Płażek *et al.*, 2014). Maintenance of turgor or its re-establishment after initial water loss is likely to sustain or increase the demand for assimilates required for cell wall deposition or protein and nucleotide biosynthesis. Although turgor maintenance is the driving force for cell expansion and thus organ growth, these processes are under metabolic control (Hare and Cress, 1997). Accordingly, *M. floridulus* showed the lowest L_RWC_ and therefore reduced turgor, indicating a lower demand for assimilates and, in relation to the increased proline accumulation in this genotype, an alternative mechanism might simultaneously maintain water balance while ensuring the continuation of metabolic processes. Proline protects the photosynthetic apparatus by functioning as an oxygen radical scavenger (Hasanuzzaman *et al.*, 2013) and as an electron sink under stress conditions (Sharma and Dietz, 2006), while its accumulation buffers cytosolic pH and maintains cell redox status (reviewed in Szabados and Savouré, 2010; Hayat *et al.*, 2012).

Soluble sugars increased in *Miscanthus* plants and proline accumulation increased significantly in *M. floridulus* and non-significantly in *M. × giganteus* growing under S and S+D combined stress, possibly to protect the membranes from lipid peroxidation as the MDA content is often used as a marker to assess the severity of oxidative stress and the degree of plant sensitivity. Similar results in *M. × giganteus* under 60 mm NaCl were observed in a previous experiment (Stavridou *et al.*, 2016) and in concentrations <100 mm NaCl (Płażek *et al.*, 2014). However, drought-induced accumulation of proline in *M. × giganteus* was also observed by Ings *et al.* (2013). The capacity for proline accumulation is species-specific and it is likely that it contributes to stress tolerance; however, it is not a prerequisite for adaptation to extreme environmental stresses (Szabados and Savouré, 2010). Despite the yield penalty imposed by the stress treatments, *M. × giganteus* and *M. floridulus* produced more M_D_ compared with the *M. sinensis* genotypes. This might be explained by the increase in proline content in combination with soluble sugars and ion accumulation, which reduces leaf osmotic potential, allowing plants to absorb more water and maintain turgor.

The reductions in photosynthesis were not severe enough to trigger reductions in yield under water deficit. Also, under moderate salinity the induced reductions in transpiration, photosynthetic efficiency, relative chlorophyll content and assimilation efficiency were not reflected in the dry biomass reduction (*M. × giganteus*) or maintenance (*M. floridulus*, *M. sin.* 1, *M. sin.* 2). In the S+D combined stress, the reductions in photosynthetic efficiency and assimilation rates were mainly due to metabolic limitations and were reflected in the reductions in dry biomass in *M. × giganteus* and *M. floridulus*. The reduction in plant growth resulting from the imposition of severe stresses is in part related to changes in whole-plant carbon status (i.e. partitioning of assimilates between different organs) and also the balance between photosynthesis and respiration (Flexas *et al.*, 2006). The more sensitive response of assimilation capacity in ambient light to the applied stress treatments indicates a possible adaptation mechanism the plants acquired in glasshouse conditions by regulating their stomata. Nevertheless, the potential *A*_sat_ achievable by the plants was affected by severe S+D combined stress and slightly by S, but not by D. The saturating intracellular CO_2_ (*A*_max_) was also reduced under S and S+D combined stress. Therefore, it is probable that the salt-induced ionic effects on photosynthetic metabolic process rather the salinity-induced osmotic stress on the photosynthetic machinery and metabolism inhibited the CO_2_ assimilation rates. Similar results of reduced net photosynthesis (*A*) under salinity were observed in 14 genetic lines of barley (Jiang *et al.*, 2006) and in maize (Stepien and Klobus, 2005).

Inhibition of photosynthesis may be caused by stomatal and/or non-stomatal limitations (Farquhar and Sharkey, 1982). Here, genotypic differences in stomatal limitation were observed between *M. × giganteus* and *M. sin.* 2, which had higher stomatal limitation compared with *M. sin*. 1 and *M. floridulus*, in which the limitation in assimilation was due to inhibition of CO_2_ metabolism. This indicates that *L*s was not the only source of the stress-induced reduction in the assimilation rate. The reduction in *C*_i_/*C*_a_ suggests that, regardless of the genotypic variation observed, under the single stresses of moderate S (5.44 dS m^−1^) and D (15 %), the limitation in photosynthesis was by diffusional restrictions on the uptake of CO_2_, most likely as a result of closed stomata (Ghannoum, 2009; Jiao *et al.*, 2017), whereas the non-significant reduction in photosynthesis under S+D combined stress was due to metabolic limitations compared with non-stressed plants.

The initial slope of the *A*/*C*_i_ response reflects the *in vivo* capacity for PEP carboxylation (*V*_pmax_) (von Caemmerer, 2000). The value of *A* would only decrease after the decreased *g*_s_ lowered *C*_i_ below the transition point of the *A*/*C*_i_ response from PEP regeneration limitation to PEP carboxylation limitation (Glowacka *et al.*, 2016). In this study *V*_pmax_ was reduced in *M. × giganteus* under S and S+D combined stress and in *M. floridulus* under all stress treatments, suggesting that the reduction in *A* is possibly induced due to reductions in PEP carboxylase activity. The non-significant reduction in *V*_pmax_ of *M. sinensis* genotypes supports the evidence that the main limitations in photosynthesis are a result of metabolic limitations. It is notable that *V*_pmax_ values did not differ between *M. sinensis* genotypes under S and S+D, indicating that these genotypes maintained their carboxylation capacity at low levels and in combination with the low *g*_s_ they maintained *A* under stress treatments.

Reduction in the *in situ* dark-adapted PSII maximum quantum efficiency (*F*_v_/*F*_m_) in *M. × giganteus* and *M. floridulus* indicated that photochemical conversion efficiency of PSII was more susceptible under stresses involving accumulated salinity (S and S+D), something that was not observed in *M. sinensis* genotypes. This is consistent with salinity studies on *M. sinensis* (Sun *et al.*, 2014), suggesting a tolerance mechanism that maintains the photosynthetic efficiency of the plant. The unaffected *F*_v_/*F*_m_ under D treatment reflects a stable conversion efficiency of PSII as *Miscanthus* may have developed the ability to effectively adapt to dry conditions, as has been previously observed in sunflower leaves (Tezara *et al.*, 1999) and in C4 grasses (Ghannoum *et al.*, 2003). It is interesting that PI decreased moderately after day 28 when plants were grown in D and more extensively in S and S+D combined stress. The PI values were lower in *M. floridulus* than in other genotypes. This demonstrates that PI, expressing the accumulation of all responses of the photosynthetic apparatus, is much more sensitive than *F*_v_/*F*_m_ in response to environmental stress (van Heerden *et al.* 2003).

The φPSII/φCO_2_ ratio has been suggested to be an effective measure of the relationship between linear electron transport and CO_2_ assimilation in leaves (Genty *et al.*, 1989; Edwards and Baker, 1993). In this study no genotypic effect was observed; however, a treatment effect was apparent over time. Regardless of the lower φPSII/φCO_2_ ratio under stress compared with non-stressed plants, it was maintained over time and the values for D (14), S (15.3) and S+D (15.3) treatments were still above those recorded in maize (~12) (Edwards and Baker, 1993), demonstrating a rate of electron transport through PSII in considerable excess of that required to sustain the observed rate of CO_2_ assimilation. Nonetheless, it has been suggested that if the φPSII/φCO_2_ in stressed leaves is considerably lower than that found in non-stressed leaves, then sinks other than CO_2_ assimilation for the products of electron transport may be operating (Fryer *et al.*, 1995). Differences in the optical properties of the leaves at different times could result in differences in φPSII/φCO_2_, which in this study is further supported, at least for the S+D combined stress, by the decline in light absorbance over time. The relatively constant ratio of φPSII/φCO_2_ with marked changes in assimilation rate under the range of the stress treatments studied suggests no stress-induced sinks for electrons outside of carbon assimilation, and consequently PSII activity is closely linked to CO_2_ fixation (Oberhuber and Edwards, 1993). It has also been suggested that the linear relationship between φPSII and φCO_2_ in C4 species is presumably due to minimal photorespiration (a sink for PSII electrons), which was confirmed in this study, as respiration in light (*R*_light_) was not responsive to the treatments (Oberhuber and Edwards, 1993). Photorespiration rates in C4 plants have been reported to be maintained under drought stress (Carmo-Silva *et al.*, 2008). The CO_2_ compensation point was not affected by the stress treatments, supporting the evidence that photorespiration was low and not sufficient to explain the decrease observed in net CO_2_ assimilation rate under the stress conditions, reflecting the effective CO_2_-concentrating mechanisms operating in *Miscanthus*. Similar observations in C4 plants under drought were made by Carmo-Silva *et al.* (2008), whereas different observations were made in C4 grasses growing under drought (Kennedy, 1977; Ghannoum *et al.*, 2003) and salinized barley (Rawson, 1986).

The LCP, which determines the amount of light intensity when the rate of photosynthesis is zero, expresses the metabolic cost of basal metabolism and represents the capacity of crops to perform well under limited light (Bellasio and Griffiths, 2014). Stress events affecting respiration or photosynthetic capacity will readily be mirrored by the LCP (Bellasio *et al.*, 2016), as in this experiment, where LCP was reduced under all stresses, with more pronounced reduction under S stress. Since there was no treatment effect on light or dark respiration, the LCP reflects the effects of stresses on photosynthetic capacity.

Dedicated biomass crops show high variability in ash and mineral content because it depends on genetic and environmental factors (Casler and Boe, 2003) as well as morphophysiological differences (Lewandowski and Schmidt, 2006; Monti *et al.*, 2008). Here, the ash content was higher in plants growing under moderate S and S+D combined stress compared with C and D conditions, with leaves having higher ash content compared with stems. The genotypic differences are possible evidence of different mechanisms of ion accumulation during treatments involving S. Biomass elements, including K, Na, Cl, P and *C*_a_, are involved in potentially severe ash deposition problems at high or moderate combustion temperatures. Chlorine especially is a major factor in ash formation and facilitates the mobility of many inorganic compounds (Jenkins *et al*., 1998; Shao *et al*., 2012). Therefore, here we used ash content as a proxy indicator of elemental content, as shown previously by Das *et al*. (2004) and Jenkins *et al*. (1996, 1998). *Miscanthus floridulus* showed increased leaf ash only under moderate S, possibly due to the dramatic decrease in stomatal conductance and transpiration, which probably drove the accumulation of minerals in the leaves and led to lower amounts of ash in the stems. Increased leaf ash was accumulated by *M. sin*. 1 and *M. × giganteus* under both stresses involving S. Conversely, *M. sin.* 2 accumulated ash in the stems under S and S+D, possibly by maintaining stable low *g*_s_, which might reflect a mechanism of compartmentalizing ions in the stem to preserve the functionality of the leaves. In previous studies in *Miscanthus*, the leaves showed higher mineral concentrations and double the amount of ash compared with stems or reproductive organs (Monti *et al.*, 2008). The different soil ECp (Fig. 5) observed in plants growing under S and S+D combined stress may be attributed to different mechanisms of ion uptake in *M. × giganteus*, *M. floridulus* and *M. sin*. 1. Under S treatment, possibly due to lack of photosynthate to produce solutes or reduced transpiration, the genotypes were unable to exclude salinity as effectively. *Miscanthus × giganteus* and *M. floridulus* had similar ash content in the stems between S and S+D treatments, yet in the leaves both genotypes increased their ash content only under S treatment. However, genotype *M. sin*. 1 accumulated more ash under the S+D treatment in both stem and leaves (Fig. 6). Interestingly, in *M. sin.* 2 under S and S+D the levels of ECp were identical and the ash content accumulated in stems was higher under these stresses, but there were no differences between treatments in the leaves, reaffirming that this genotype is able to sustain its photosynthesis, growth and transpiration, albeit at a lower level compared with the other genotypes, under single S or combined S+D stress.

Production of biostock for fermentation requires a low lignin content, which in this study was not affected by the stress treatments. The lignin content in *Miscanthus* was genotype- and tissue type-specific but was not affected by D and moderate S stresses in combination or in isolation. In previous studies it was suggested that the normal lignin range for non-woody biomass was between 11 and 27 % (Bagby *et al.*, 1971). Among the genotypes studied here, lignin ranged between 11 and 20.2 % for leaves and stems, and stems had higher lignin content compared with leaves. The lack of response suggests that lignin accumulation may not have a mechanistic role in the early response to D or moderate S stresses but high lignin accumulation was associated with the slow growth phenotype of *M. sin.* 2.

### Conclusions

We examined the responses to different stress treatments of the commercial genotype *M. × giganteus* and three novel *Miscanthus* accessions. The four genotypes displayed diverse responses that may be summarized as conservative and non-conservative growth strategies (Table 11) and allowed us to test different strategies of responses to single or combined stress. The high-yielding *M. × giganteus* and *M. floridulus* genotypes produced more yield under all treatments than the slower-growing but more stress-tolerant *M. sinensis* genotypes. Therefore, on the basis of biomass accumulation, we conclude that, despite the reduction in yield under stress treatments, it is likely that the genotypes producing higher yield under single stress or non-stressed conditions would be preferred. However, the slow-growing conservative strategy may prove superior under more consistent and/or extreme stresses and combinations of stresses than those tested here.

**Table 11.** Summary of biomass, morphology, composition and photosynthetic response of four *Miscanthus* genotypes grown in control, single stress and combined salinity and water stress treatments

Genotype	Control	Drought	Salinity	Salinity + drought
*M. × giganteus*	High biomass, high *g*_s_, low ash	High biomass, low ash	High biomass, reduced chlorophyll, low T, reduced *F*_v_/*F*_m_, PI and *V*_pmax_, high lignin, high ash in leaves	High biomass, reduced chlorophyll, *F*_v_/*F*_m_ and PI, low *A* and *g*_s_, reduced *V*_pmax_, high ash in leaves
*M. floridulus*	High biomass, high T, high *g*_s_, low ash	High biomass, low T, low *A V*_pmax_ and *g*_s_, low ash	High biomass, reduced chlorophyll, low T, low *F*_v_/*F*_m_, low PI, low assimilation rate (*A*) *V*_pmax_ and *g*_s_, high proline, high ash	High biomass, increased WUE, reduced T, reduced chlorophyll, low *F*v/*F*m, low PI, reduced *A*, *V*_pmax_ and *g*_s_, reduced L_RWC_, high proline, low ash
*M. sin.* 1	Moderate biomass, high *g*_s_, low ash	Low biomass, low ash	Low biomass, reduced chlorophyll, reduced T, maintained *F*_v_/*F*_m_ and PI, high ash in leaves	Low biomass, reduced chlorophyll, reduced T, low *A* and *g*_s_, high ash in leaves
*M. sin.* 2	Low biomass, high chlorophyll, low *g*_s_, low ash, high lignin	Low biomass, low ash	Low biomass, increased chlorophyll, maintained *F*_v_/*F*_m_ and PI, high ash in stems	Low biomass, high ash in stems, reduced chlorophyll

The four genotypes in the study were the commercial standard *M. × giganteus* and three selected genotypes: *M. floridulus* and two *M. sinensis* (*M. sin*. 1 and *M. sin*. 2).

PI, performance index, *T*; total transpiration; *V*_pmax_, CO_2_ saturated phosphoenol pyruvate carboxylation rate; *g*_s_, stomatal conductance; *A*, assimilation rate; *Fv*/*F*_m_, maximum quantum yield of PSII.

Next, we considered whether there were advantages associated with the choice of high-yielding genotype. *Miscanthus × giganteus* may be categorized as an optimistic plant growing to high biomass utilizing a non-conservative growth strategy. The *M. floridulus* genotype studied here may be described as even more so by producing more above- and below-ground biomass than *M. × giganteus*, mainly due to more leaf and root production and at a lower WUE in the control treatment than *M. × giganteus*. The response of the *M. floridulus* genotype involved higher levels of leaf senescence, with significant reductions in transpiration, PI and L_RWC_. The reduction in photosynthetic efficiency and assimilation rates under stress were mainly due to metabolic limitations, and this is reflected in the reduction in M_D_ in both genotypes. However, most photosynthetic parameters were reduced more in the *M. floridulus* genotype than in *M. × giganteus*. *Miscanthus × giganteus* maintained higher assimilation rates and *V*_pmax_ irrespective of treatment and *M. floridulus* responded by more severe transpiration control and a significant increase in proline content. Thus, we conclude that the *M. floridulus* genotype is a favourable choice in adopting a less conservative growth strategy than even *M. × giganteus* in favourable growth conditions, but that it responds to stress more severely in adjusting leaf senescence, stomatal control and biochemistry. These adjustments confer greater protection on the *M. floridulus* genotype, as measured by lower MDA levels, than on *M. × giganteus*. The slightly higher transpiration rates in well-watered treatments and high ash and biomass accumulation in the *M. floridulus* genotype may make it more efficient at remediating saline soils than *M. × giganteus* if an effective use of high ash biomass is available. This would likely only be the case if the saline soils were not subject to drought stress because of the greater transpiration reduction in the *M. floridulus* genotype, which reduced ash accumulation in D treatments.

This study highlights the available diversity of novel *Miscanthus* genotypes that are resilient to combinations of stress. We identify the potential for single genotypes to outperform *M. × giganteus* in non-stressed and stressed conditions. We also highlight the different response mechanisms, some of which appear to have a general yield penalty; for example, in the stay-green genotype *M. sin.* 2 some traits, such as the accumulation of soluble sugars from the same conservative genotype, may be suitable breeding targets for transfer to genotypes with non-conservative growth strategies because such traits are unlikely to impact final biomass yield. This information is expected to contribute to a deeper fundamental knowledge of different mechanistic responses identified as suitable for further exploitation in developing resilient *Miscanthus* crops.

## SUPPLEMENTARY DATA

Supplementary data are available online at https://academic.oup.com/aob and consist of the following. Figure S1: water use efficiency and transpiration of *M.* × *giganteus*, *M. floridulus*, *M. sin.* 1 and *M. sin.* 2 in response to C, D, D and S+D treatments over the 67-d experimental period. Figure S2: ratio of intercellular to external CO_2_ concentration and ratio of stomatal conductance to intracellular CO_2_ concentration of *M.* × *giganteus*, *M. floridulus*, *M. sin.* 1 and *M. sin.* 2 under C, D, S and S+D conditions over three time points measured at 400 μmol mol^−1^ CO_2_ and 1500 μmol photons m^2^ s^−1^ in the controlled environment growth chamber. Figure S3: leaf relative water content of *M.* × *giganteus*, *M. floridulus*, *M. sin.* 1 and *M. sin.* 2 under C, D, S and S+D conditions. Figure S4: changes in soil moisture content of *M.* × *giganteus*, *M. floridulus*, *M. sin.* 1 and *M. sin.* 2 under C, D, S and S+D conditions over the 67-d experimental period. Figure S5: target weight of pots for the different genotypes (*M.* × *giganteus*, *M. floridulus*, *M. sin.* 1 and *M. sin.* 2) under the different stress conditions (C, D, S and S+D) over the 67-d experimental period. Table S1: interaction effect between treatment and genotype on accumulated dry biomass. Table S2: interaction effect between treatment and genotype on growth parameters. Table S3: Tukey HSD *post hoc* test for the three-way interaction effect between genotypes, treatments and days on height of the main stem in C, D, S and S+D conditions. Table S4: Tukey HSD *post hoc* test for the interaction effect between treatment and day for stomatal conductance. Table S5: Tukey HSD *post hoc* test for the interaction effect between treatment and time of day (pre-dawn and midday) for leaf water potential. Table S6: Tukey *post hoc* test for the interaction effects between treatment and day and between genotype and day for maximum quantum efficiency. Table S7: Tukey HSD *post hoc* test for the interaction effect between genotype, treatment and day for the performance index. Table S8: Tukey HSD *post hoc* test for the interaction effect between treatment and genotype for MDA content and soluble sugars.

mcz009_suppl_Supplementary_Material
