# Supplementary material for: Novel Miscanthus genotypes selected for different drought tolerance phenotypes show enhanced tolerance across combinations of salinity and drought treatments
Source: Ann Bot. 2019 Mar 10;124(4):653–74. doi: 10.1093/aob/mcz009 (PMC6821188; doi:10.1093/aob/mcz009)
Supplement: mcz009_suppl_Supplementary_Material [file mcz009_suppl_supplementary_material.docx]

**Supplemental material**

Novel *Miscanthus* genotypes selected for different drought tolerance phenotypes show enhanced tolerance across combinations of salinity and drought treatments

Evangelia Stavridou, Richard J. Webster and Paul. R. H. Robson

**
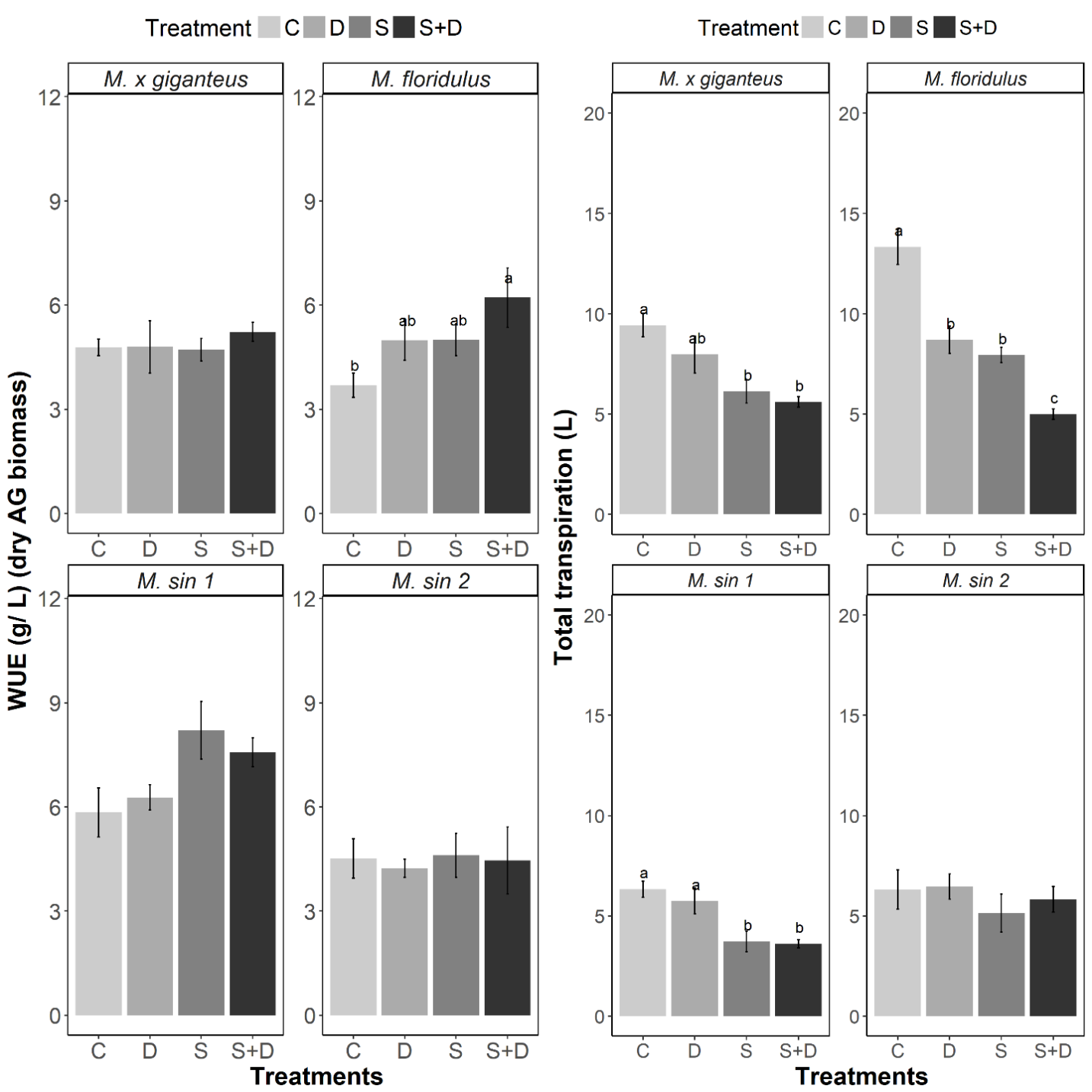
****Figure S1.** Water use efficiency (WUE) (g/L) (Left panel) and Transpiration (L) (Right panel) of *M. x giganteus*, *M. floridulus*, *M. sin* 1 and *M. sin* 2 in response to Control (C), Drought (D), Salinity (S) and Salinity and Drought (S+D) treatments over the 67-days experimental period. Data show the mean ± SE of the data (n=6).

**
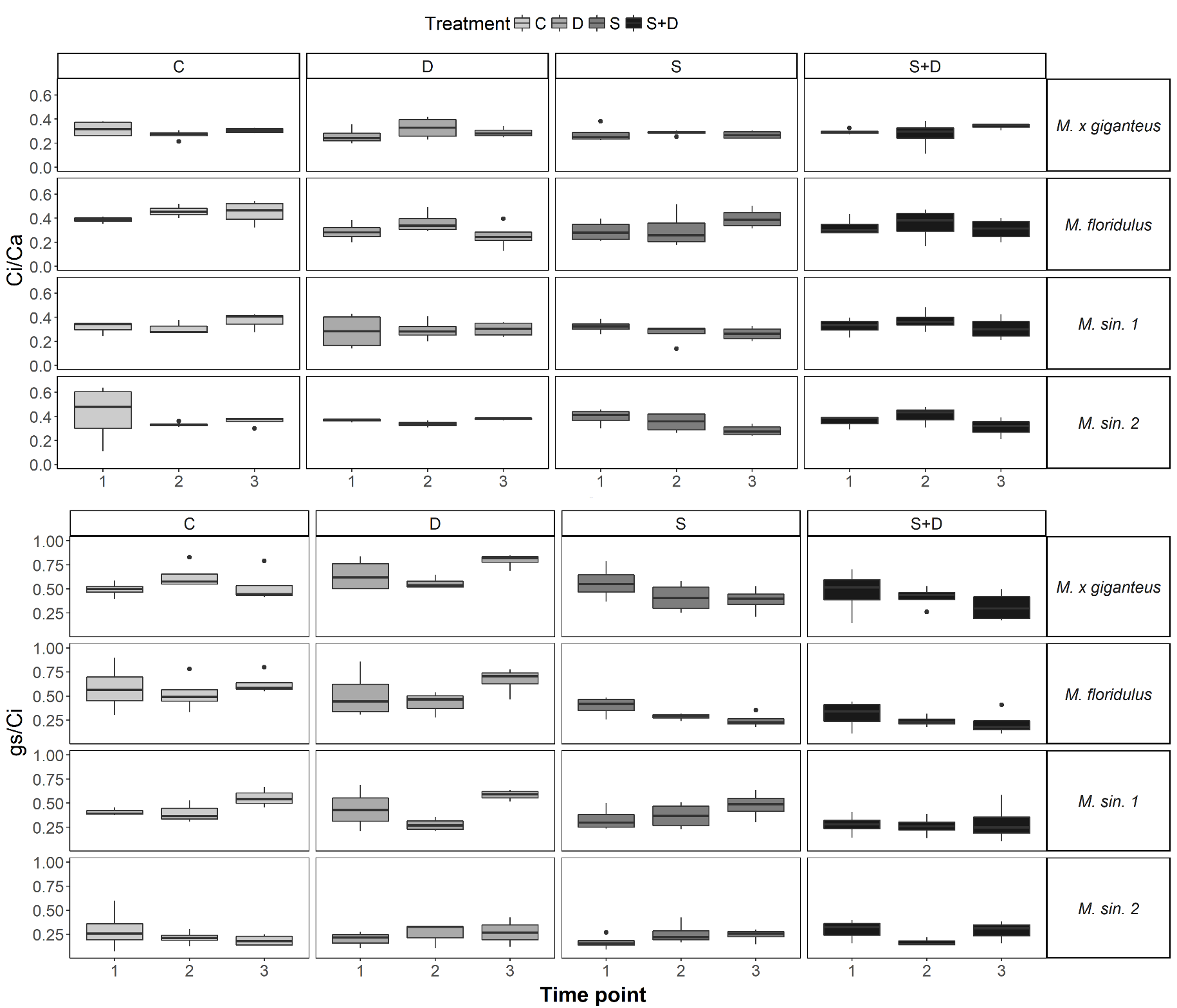
**

**Figure S2.** Ratio of intercellular to external CO_2_ concentration (Ci/Ca) (Upper panel) and ratio of stomatal conductance (*g*_s_) to intracellular CO_2_ concentration (Lower panel) of *M. x giganteus*, *M. floridulus*, *M. sin* 1 and *M. sin* 2 at C, D, S and S+D conditions over three time-points (Weeks: 2, 4 and 8) measured at 400 μmol mol^-1^ CO_2_, 1500 μmol photon m^2^ s^-1^ in the controlled environment growth chamber. Data are mean ± Standard Error (n= 4).


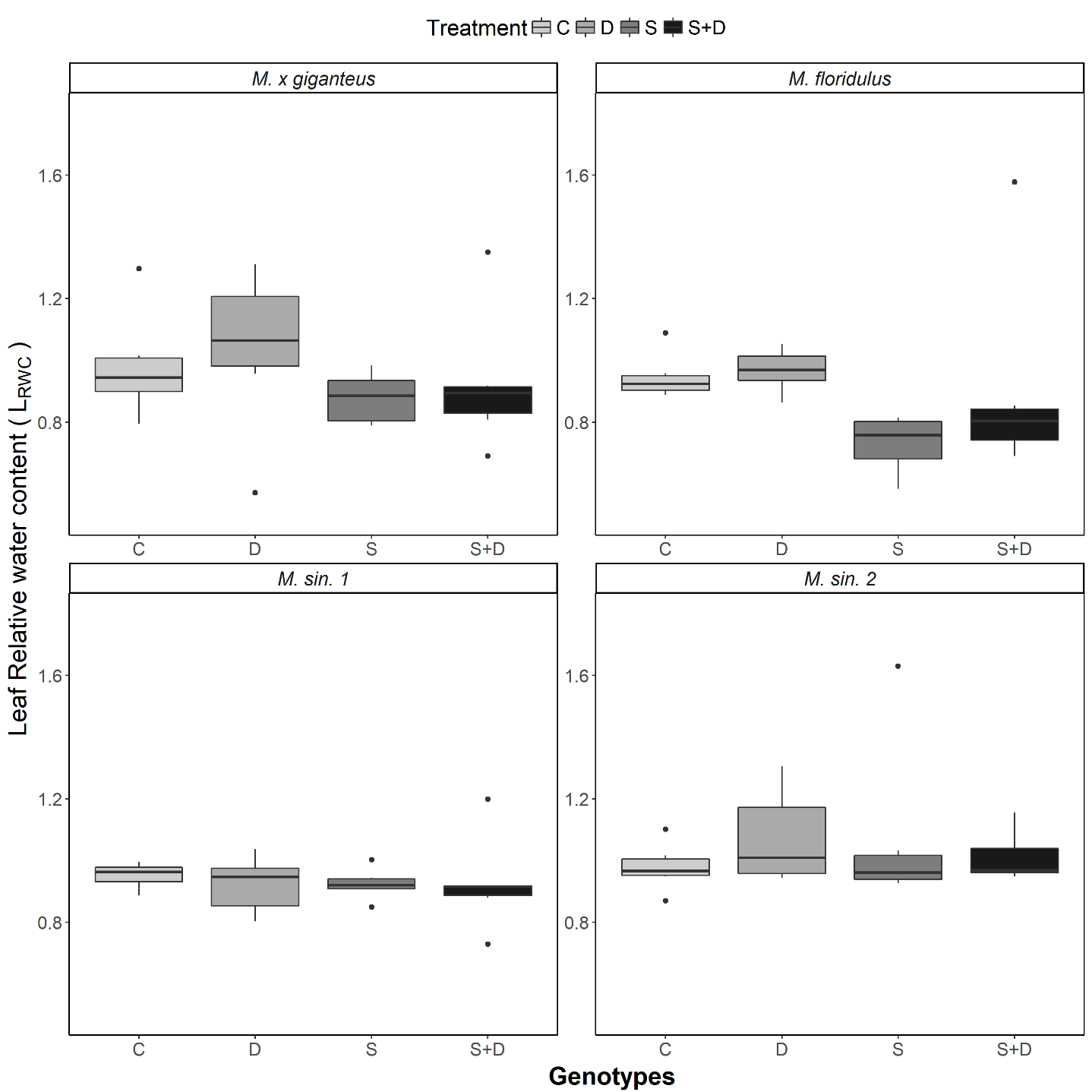


**Figure S3.** Leaf relative water content (L_RWC_) of *M. x giganteus*, *M. floridulus*, *M. sin.* 1 and *M. sin.* 2 at C, D, S and S+D conditions. Data show the median (2nd quartile; horizontal line) and the 1st and 3rd quartiles of the data. Dots indicate outliers; (n=6).

**
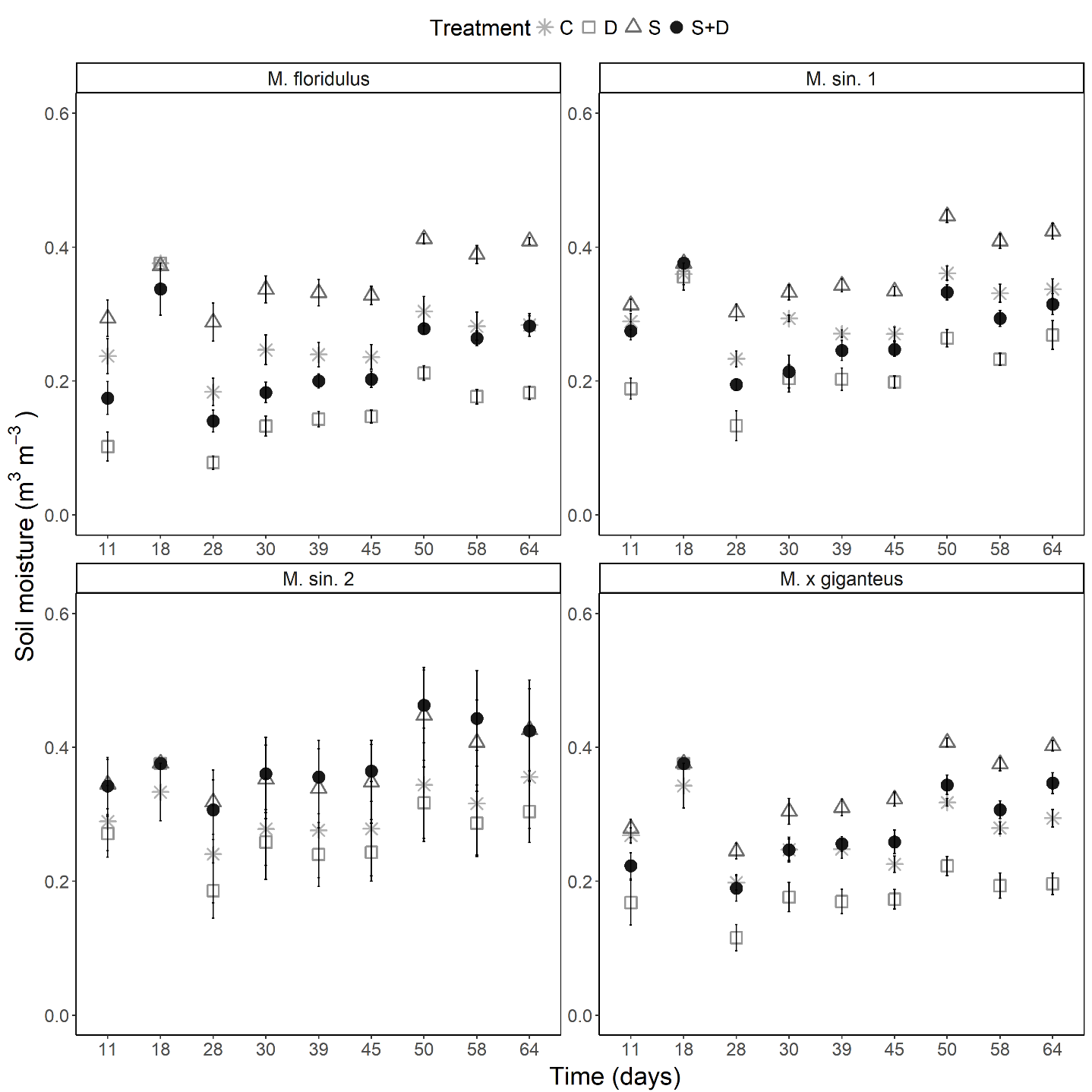
**

**Figure S4.** Changes in soil moisture content (m^3^ m^-3^) of *M. x giganteus*, *M. floridulus*, *M. sin.* 1 and *M. sin.* 2 at C, D, S and S+D conditions over the 67-days experimental period. Data show the mean ± SE; n=6.


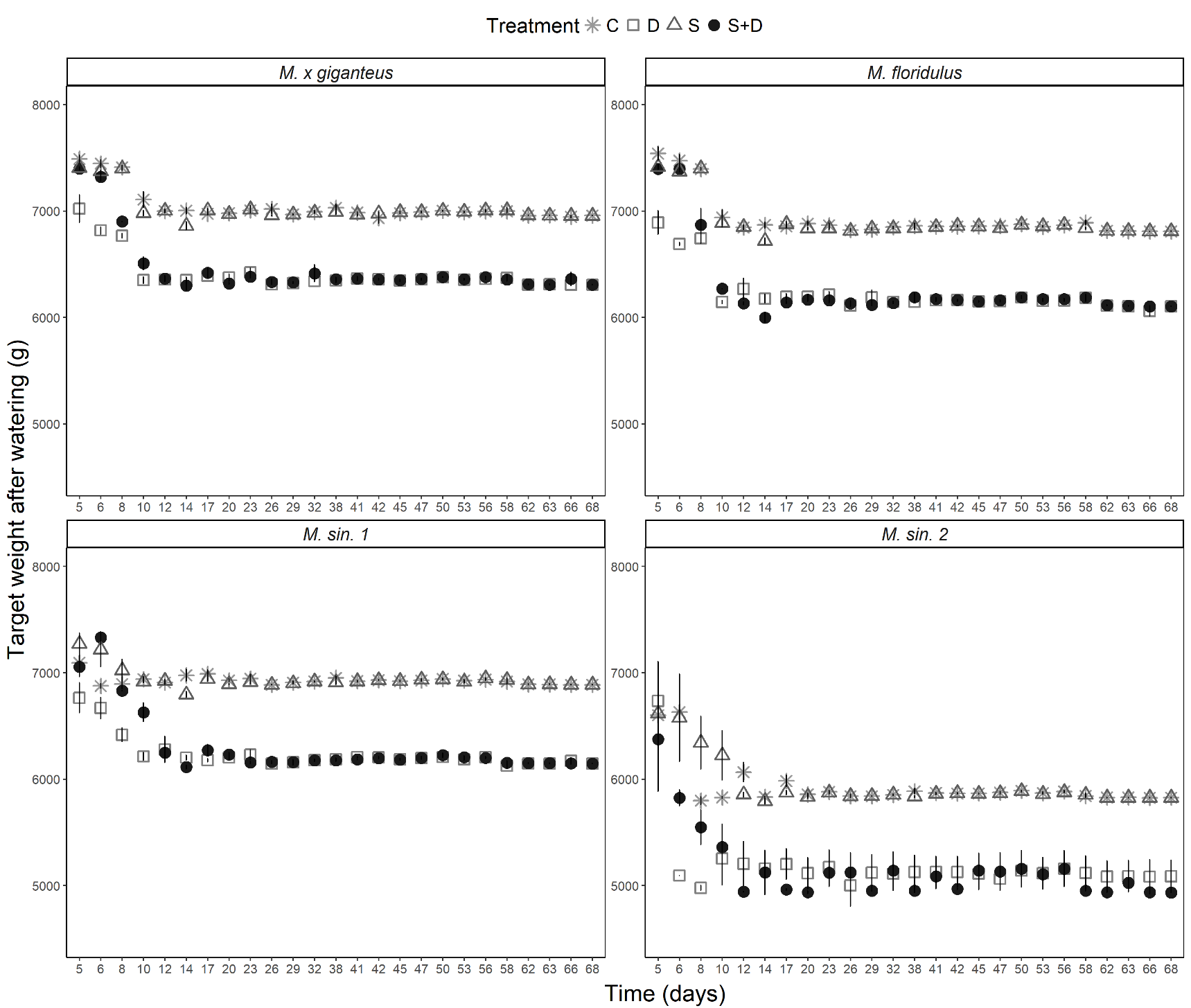


**Figure S5.** Target weight of pots for the different genotypes (*M. x giganteus*, *M. floridulus*, *M. sin.* 1 and *M. sin.* 2) under the different stress conditions (C, D, S and S+D) over the 67-days experimental period. Data show the mean ± SE; n=6.

**Table S1.** **Interaction effect between treatment and genotype on the accumulated dry biomass.** Average values and Tukey HSD (HSD) *post-hoc* test for the interaction between treatment and genotype on the Above-ground dry matter (AG **M_D_**), Leaf dry matter (L_D_), Stem dry matter (S_D_), Below-ground **M_D_** (BG **M_D_**), Rhizome **M_D_** (RZ **M_D_**), Root dry matter (R_D_) and Total **M_D_**.

| **Treatment** | **Genotypes** | **AG M_D_** | **_HSD_** | **L_D_** | **_HSD_** | **S_D_** | **_HSD_** | **BG M_D_** | **_HSD_** | **RZ M_D_** | **_HSD_** | **R_D_** | **_HSD_** | **Total M_D_** | **_HSD_** |
| --- | --- | --- | --- | --- | --- | --- | --- | --- | --- | --- | --- | --- | --- | --- | --- |
| **C** | *M. x giganteus* | 44.78±2.55 | a | 14.125±0.93 | a | 30.65±2.69 | ab | 70.27±28.68 | ab | 52.08±5.23 | a | 18.19±1.41 | ab | 115.06±8.39 | ab |
|  | *M. fluoridulus* | 48.87±5.62 | a | 17.02±1.88 | a | 31.85±3.81 | a | 86.89±35.47 | a | 49.95±3.75 | a | 36.94±12.09 | a | 135.76±20.47 | a |
|  | *M. sin.* 1 | 35.81±2.29 | ab | 14.81±1.06 | a | 21.00±1.39 | bc | 40.7±16.61 | b | 32.32±5.535 | b | 8.37±0.63 | b | 76.51±6.76 | bc |
|  | *M. sin.* 2 | 26.3±2.43 | b | 14.21±1.63 | a | 12.09±0.96 | c | 37.63±15.36 | b | 20.08±1.93 | b | 17.55±1.97 | ab | 63.93±5.98 | c |
| **D** | *M. x giganteus* | 36.25±4.23 | a | 11.31±1.35 | a | 24.94±2.99 | a | 70.27±6.15 | ab | 46.42±3.39 | a | 22.06±1.46 | ab | 104.73±8.32 | ab |
|  | *M. fluoridulus* | 42.21±3.52 | a | 14.21±1.18 | a | 28±2.55 | a | 86.89±15.00 | a | 50.72±3.721 | a | 31.7±4.13 | a | 124.62±8.67 | a |
|  | *M. sin.* 1 | 36.26±4.41 | a | 15.26±1.78 | a | 21.01±2.63 | ab | 40.7±5.94 | bc | 26.57±3.57 | b | 11.46±2.01 | b | 74.3±8.67 | b |
|  | *M. sin.* 2 | 27.6±3.28 | a | 15.3±1.78 | a | 12.3±1.51 | b | 37.63±3.75 | c | 22.24±3.72 | b | 27.01±5.92 | a | 76.85±12.00 | b |
| **S** | *M. x giganteus* | 28.67±3.37 | a | 8.71±1.03 | b | 19.96±2.40 | ab | 59.74±6.56 | a | 45.02±5.17 | a | 14.72±1.51 | ab | 88.42±9.196 | a |
|  | *M. fluoridulus* | 39.51± 4.00 | ab | 14.06±1.37 | a | 25.44±2.69 | a | 67.85±4.30 | a | 47.07±2.79 | a | 20.77±1.88 | a | 107.36±7.88 | a |
|  | *M. sin.* 1 | 28.76±1.77 | ab | 11.52±0.62 | ab | 17.23±1.36 | bc | 29.95±2.07 | b | 24.01±1.95 | b | 5.94±0.44 | c | 58.71±3.47 | b |
|  | *M. sin.* 2 | 21.5±2.88 | b | 11.03±1.72 | ab | 10.46±1.35 | c | 30.47±3.47 | b | 16.54±1.28 | b | 13.93±2.39 | b | 51.97±6.31 | b |
| **S+D** | *M. x giganteus* | 29.25±1.96 | a | 9.54±0.31 | b | 19.71±1.83 | a | 56.56±2.34 | a | 40.16±2.47 | a | 16.41±0.97 | a | 85.82±2.79 | a |
|  | *M. fluoridulus* | 30.81±4.00 | a | 9.86±1.05 | ab | 20.95±2.97 | a | 50.62±2.96 | a | 33.04±2.24 | a | 17.57±1.23 | a | 81.42±6.87 | a |
|  | *M. sin.* 1 | 27.11±1.56 | a | 11.76±0.82 | ab | 15.35±0.90 | ab | 27.38±2.21 | b | 20.82±1.83 | b | 6.56±0.45 | b | 54.5±3.64 | b |
|  | *M. sin.* 2 | 23.51±1.99 | a | 13.16±1.14 | a | 10.35±1.03 | b | 34.46±2.78 | b | 17.89±1.344 | b | 16.57±2.10 | a | 57.14±4.52 | b |

Data are mean ±Standard Error (n=6).

Different letters indicate significant differences at p<0.05.

**Table S2. Interaction effect between treatment and genotype on the growth parameters** Average values and Tukey HSD (HSD) *post-hoc* test for the interaction between treatment and genotype on the morphological data of Height (cm), Leaf number of the main stem, total leaf number, leaf area (L_A,_ cm^2^) and Stem number. Different letters indicate significant differences between treatments for each genotype (p<0.05).

| **Treatment** | **Genotypes** | **Height** | **_HSD_** | **Leaf number** | **_HSD_** | **Total Leaf number** | **_HSD_** | **L_A_** | **_HSD_** | **Stem number** | **_HSD_** |
| --- | --- | --- | --- | --- | --- | --- | --- | --- | --- | --- | --- |
| **C** | *M. x giganteus* | 137±55.93 | a | 15±6.12 | b | 17.55±7.96 | b | 17.55±7.96 | b | 5.33±0.92 | a |
|  | *M. fluoridulus* | 114.33±2.67 | a | 23.16±3.32 | a | 82.26±6.76 | b | 82.26±6.76 | a | 5.5±0.85 | a |
|  | *M. sin.* 1 | 80.66±4.95 | b | 13.33±0.49 | bc | 37.54±5.64 | b | 37.54±5.64 | b | 8.16±0.94 | a |
|  | *M. sin.* 2 | 27.33±1.54 | c | 10.5±0.43 | c | 82.96±5.95 | a | 82.96±5.95 | a | 5.66±0.49 | a |
| **D** | *M. x giganteus* | 123.5±8.38 | a | 14.16±5.78 | b | 87.34±18.85 | a | 87.34±18.85 | a | 4.83±1.11 | a |
|  | *M. fluoridulus* | 98.5±3.48 | b | 23.16±1.79 | a | 101.36±8.16 | a | 101.36±8.16 | a | 5.66±1.28 | a |
|  | *M. sin.* 1 | 75.08±6.00 | c | 14.33±0.88 | b | 38.13±5.43 | b | 38.12±5.43 | b | 6.5±0.43 | a |
|  | *M. sin.* 2 | 27.83±1.47 | d | 11.5±0.43 | b | 86.86±9.14 | a | 86.86±9.14 | a | 6±0.52 | a |
| **S** | *M. x giganteus* | 111.08±3.81 | a | 15.16±0.31 | a | 58.18±23.75 | ab | 58.18±18.75 | ab | 4.16±0.31 | a |
|  | *M. fluoridulus* | 93.83±4.24 | b | 15.66±1.76 | a | 91.85±8.61 | a | 91.85±8.612 | a | 5.83±0.70 | a |
|  | *M. sin.* 1 | 70.92±3.49 | c | 13.83±0.60 | a | 39.45±7.87 | b | 39.45±7.87 | b | 6.16±0.60 | a |
|  | *M. sin.* 2 | 28.08±1.63 | d | 10.5±0.56 | b | 88.91±7.25 | a | 88.91±7.25 | a | 4±0.68 | a |
| **S+D** | *M. x giganteus* | 107.33±5.39 | a | 14.33±0.95 | a | 78.56±14.05 | ab | 78.56±14.05 | ab | 5.5±1.33 | a |
|  | *M. fluoridulus* | 89.66±4.02 | b | 17.33±2.78 | a | 108.62±12.86 | a | 108.62±12.86 | a | 5±1.15 | a |
|  | *M. sin.* 1 | 67.08±3.19 | c | 13.5±0.43 | ab | 53.62±6.62 | b | 53.62±6.62 | b | 5.16±0.40 | a |
|  | *M. sin.* 2 | 28.25±1.36 | d | 9.83±0.60 | b | 59.93±8.22 | b | 59.93±8.22 | b | 6±0.57 | a |

Data are mean ±Standard Error (n=6).

Different letters indicate significant differences at p<0.05.

**Table S3.** Tukey HSD (_HSD_) *post-hoc* test for the 3-way interaction effect between genotypes, treatments and days on height of the main stem (cm) in Control (C), Drought (D), Salinity (S) and Combined (S+D) stress conditions. Different letters indicate significant differences between days for each genotype and treatment (p<0.05).

| **Treatment** | **Day** | ***M. x giganteus*** | | ***M. sin 1*** | | ***M. sin 2*** | | ***M. floridulus*** | |
| --- | --- | --- | --- | --- | --- | --- | --- | --- | --- |
|  |  | **Height** | **_HSD_** | **Height** | **_HSD_** | **Height** | **_HSD_** | **Height** | **_HSD_** |
| **C** | **5** | 96.8 ± 6.91 | b | 46.5 ± 3.27 | c | 25.3 ± 1.92 | a | 87 ± 4.56 | d |
|  | **14** | 101.4 ± 6.38 | ab | 49.6 ± 3.42 | c | 25.6 ± 1.89 | a | 94 ± 4.03 | cd |
|  | **20** | 103.2 ± 6.49 | ab | 51.4 ± 3.39 | c | 26.2 ± 1.78 | a | 96.2 ± 3.98 | bcd |
|  | **29** | 105.7 ± 6.50 | ab | 54.6 ± 3.60 | c | 26.1 ± 1.72 | a | 99.5 ± 3.64 | abcd |
|  | **40** | 107.6 ± 6.98 | ab | 57.8 ± 3.73 | bc | 26.4 ± 1.75 | a | 102.4 ± 3.70 | abcd |
|  | **49** | 112 ± 7.90 | ab | 60.6 ± 3.62 | bc | 26.4 ± 1.63 | a | 106.2 ± 3.10 | abc |
|  | **54** | 120.8 ± 10.25 | ab | 63.3 ± 3.87 | abc | 26.8 ± 1.60 | a | 107.8 ± 3.15 | abc |
|  | **64** | 129.2 ± 11.29 | ab | 72.8 ± 4.84 | ab | 27.3 ± 1.43 | a | 112.3 ± 2.64 | ab |
|  | **68** | 137 ± 12.54 | a | 80.6 ± 4.95 | a | 27.3 ± 1.54 | a | 114.3 ± 2.67 | a |
| **D** | **5** | 96.2 ± 6.72 | a | 46.3 ± 2.77 | c | 26 ± 1.46 | a | 86.42 ± 3.74 | a |
|  | **14** | 100.8 ± 6.33 | a | 49.8 ± 3.15 | bc | 26.3 ± 1.55 | a | 90.3 ± 3.68 | a |
|  | **20** | 101.6 ± 6.07 | a | 51.8 ± 3.33 | bc | 26.4 ± 1.44 | a | 90.9 ± 3.57 | a |
|  | **29** | 103.3 ± 6.32 | a | 53.8 ± 3.60 | bc | 27 ± 1.18 | a | 92.6 ± 3.61 | a |
|  | **40** | 104.9 ± 6.24 | a | 56.8 ± 3.85 | abc | 27.6 ± 1.43 | a | 94.6 ± 3.00 | a |
|  | **49** | 107.2 ± 6.01 | a | 58.2 ± 4.30 | abc | 27.8 ± 1.40 | a | 96 ± 3.066 | a |
|  | **54** | 112.3 ± 7.24 | a | 61.2 ± 4.24 | abc | 27.5 ± 1.54 | a | 97.3 ± 3.09 | a |
|  | **64** | 118.3 ± 7.88 | a | 67.8 ± 4.81 | ab | 27.8 ± 1.57 | a | 98 ± 3.21 | a |
|  | **68** | 123.5 ± 8.38 | a | 75.1 ± 6.00 | a | 27.8 ± 1.47 | a | 98.5 ± 3.48 | a |
| **S** | **5** | 97.2 ± 6.36 | a | 47.6 ± 2.78 | d | 26.8 ± 1.75 | a | 86.3 ± 4.13 | a |
|  | **14** | 101.9 ± 5.94 | a | 52 ± 3.08 | cd | 27.5 ± 1.68 | a | 90.8 ± 4.21 | a |
|  | **20** | 103 ± 5.91 | a | 53.7 ± 2.92 | bcd | 27.5 ± 1.68 | a | 92.3 ± 4.26 | a |
|  | **29** | 102.9 ± 5.88 | a | 55.3 ± 3.02 | bcd | 27.6 ± 1.64 | a | 92.6 ± 4.32 | a |
|  | **40** | 104.4 ± 5.74 | a | 58.2 ± 3.18 | abcd | 28 ± 1.59 | a | 94 ± 4.38 | a |
|  | **49** | 105.3 ± 5.65 | a | 59.8 ± 2.48 | abcd | 28 ± 1.59 | a | 94 ± 4.17 | a |
|  | **54** | 105.1 ± 5.76 | a | 62.4 ± 2.87 | abc | 28.1 ± 1.51 | a | 94.5 ± 4.14 | a |
|  | **64** | 107.5 ± 5.43 | a | 66.2 ± 3.19 | ab | 28.25 ± 1.37 | a | 93.8 ± 4.19 | a |
|  | **68** | 111.1 ± 3.81 | a | 70.9 ± 3.48 | a | 28.5 ± 1.45 | a | 93.8 ± 4.24 | a |
| **S+D** | **5** | 95.3 ± 6.65 | a | 47.5 ± 3.02 | c | 26.3 ± 1.49 | a | 85.6 ± 4.36 | a |
|  | **14** | 100.3 ± 6.27 | a | 51.2 ± 2.36 | bc | 26.5 ± 1.43 | a | 88.1 ± 3.99 | a |
|  | **20** | 100.3 ± 6.03 | a | 53.2 ± 2.25 | bc | 27.25 ± 1.45 | a | 88.3 ± 4.15 | a |
|  | **29** | 100.8 ± 6.02 | a | 54.8 ± 2.62 | bc | 26.9 ± 1.48 | a | 88.9 ± 3.85 | a |
|  | **40** | 101.5 ± 6.02 | a | 56.7 ± 2.63 | abc | 27.2 ± 1.47 | a | 89.3 ± 3.76 | a |
|  | **49** | 97.3 ± 8.93 | a | 58 ± 2.57 | abc | 27.5 ± 1.50 | a | 89.8 ± 4.31 | a |
|  | **54** | 102.5 ± 6.19 | a | 60.8 ± 2.42 | ab | 27.5 ± 1.38 | a | 89.9 ± 4.22 | a |
|  | **64** | 104.8 ± 5.55 | a | 62.3 ± 2.48 | ab | 27.6 ± 1.52 | a | 89.8 ± 3.99 | a |
|  | **68** | 107.3 ± 5.38 | a | 67.1 ± 3.19 | a | 27.75 ± 1.54 | a | 89.6 ± 4.02 | a |

Data are mean ±Standard Error (n=6).

Different letters indicate significant differences at p<0.05.

**Table S4.** Tukey HSD (_HSD_) *post-hoc* test for the interaction effect between treatment and day for the stomatal conductance (*g*_s_, mmol m^-2^ s^-1^). Different lowercase letters indicate significant differences between days for each genotype (p<0.05).

| **Day** | **C** | | **D** | | **S** | | **D+S** | |
| --- | --- | --- | --- | --- | --- | --- | --- | --- |
|  | ***g*s** | **_HSD_** | ***g*_s_** | **_HSD_** | ***g*_s_** | **_HSD_** | ***g*_s_** | **_HSD_** |
| **11** | 156.9 ± 17.45 | a | 109.3 ± 11.93 | a | 118.5 ± 7.81 | a | 94.1 ± 9.99 | a |
| **17** | 103.7 ± 7.46 | b | 82.6 ± 9.12 | a | 98.5 ± 8.79 | ab | 69.7 ± 7.60 | ab |
| **25** | 111.7 ± 9.64 | ab | 97.75 ± 8.00 | a | 96.7 ± 8.75 | ab | 86.6 ± 7.52 | a |
| **33** | 107.4 ± 6.06 | ab | 95.1 ± 9.52 | a | 74.6 ± 5.31 | bcd | 66.7 ± 6.01 | ab |
| **39** | 107.2 ± 5.77 | ab | 94.9 ± 9.55 | a | 87.5 ± 5.45 | abc | 65.8 ± 5.26 | ab |
| **49** | 116.4 ± 9.93 | ab | 96.4 ± 10.17 | a | 66.8 ± 4.45 | cd | 49.9 ± 3.74 | b |
| **55** | 106.3 ± 11.75 | b | 93.3 ± 9.88 | a | 54.1 ± 4.81 | d | 50.7 ± 5.78 | b |
| **62** | 92.0 ± 7.88 | b | 74.3 ± 7.43 | a | 59.8 ± 3.68 | d | 46.3 ± 4.51 | b |

Data are mean ±Standard Error (n=24).

Different letters indicate significant differences at p<0.05.

**Table S5.** Tukey HSD (_HSD_) *post-hoc* test for the interaction effect between treatment and time of the day (pre-dawn and midday) for the leaf water potential (Ψ_leaf_). Different uppercase letters indicate significant differences within treatments (WT) for each time of the day at p<0.05. Different lowercase letters indicate significant differences between treatments (BT) at the two-time points of the day (p<0.05).

| **Treatment** | **Pre-dawn** | | | **Midday** | | |
| --- | --- | --- | --- | --- | --- | --- |
|  | **Ψ_leaf_** | **_HSD-BT_** | **_HSD-WT_** | **Ψ_leaf_** | **_HSD-BT_** | **_HSD-WT_** |
| **C** | -2.27 ± 0.077 | ab | B | -1.87 ± 0.111 | a | A |
| **D** | -2.24 ± 0.138 | a | A | -2.39 ± 0.175 | b | A |
| **S** | -2.83 ± 0.162 | b | B | -2.25 ± 0.104 | ab | A |
| **S+D** | -2.52 ± 0.151 | ab | A | -2.37 ± 0.128 | b | A |
| **Total** | -2.465 | b | | -2.219 | a | |

Data are mean ±Standard Error (n=24).

Different letters indicate significant differences at p<0.05.

**Table S6.** Tukey HSD (_HSD_) *post-hoc* test for the interaction effect between treatment and day and genotype and day for the maximum quantum efficiency (*F*_v_/*F*_m_). Different lowercase letters indicate significant differences between days for each treatment (p<0.05).

| **Day** | **C** | | **D** | | **S** | | **S+D** | |
| --- | --- | --- | --- | --- | --- | --- | --- | --- |
|  | ***F*_v_/*F*_m_** | **_HSD_** | ***F*_v_/*F*_m_** | **_HSD_** | ***F*_v_/*F*_m_** | **_HSD_** | ***F*_v_/*F*_m_** | **_HSD_** |
| **11** | 0.768 ± 0.003 | ab | 0.760 ± 0.009 | abc | 0.772 ± 0.003 | a | 0.759 ± 0.007 | a |
| **19** | 0.775 ± 0.003 | a | 0.767 ± 0.004 | ab | 0.766 ± 0.003 | ab | 0.76 ± 0.004 | a |
| **28** | 0.745 ± 0.004 | c | 0.741 ± 0.004 | c | 0.74 ± 0.004 | cd | 0.740 ± 0.005 | abc |
| **33** | 0.749 ± 0.003 | c | 0.752 ± 0.004 | abc | 0.745 ± 0.004 | bcd | 0.744 ± 0.005 | ab |
| **47** | 0.752 ± 0.006 | bc | 0.752 ± 0.005 | abc | 0.724 ± 0.006 | d | 0.719 ± 0.009 | bc |
| **55** | 0.775 ± 0.005 | a | 0.775 ± 0.004 | a | 0.752 ± 0.006 | abc | 0.748 ± 0.005 | ab |
| **61** | 0.761 ± 0.006 | abc | 0.747 ± 0.007 | bc | 0.730 ± 0.009 | cd | 0.715 ± 0.009 | c |
| **Day** | ***M.* x *giganteus*** | | ***M. sin 1*** | | ***M. sin 2*** | | ***M. floridulus*** | |
|  | ***F*_v_/*F*_m_** | **_HSD_** | ***F*_v_/*F*_m_** | **_HSD_** | ***F*_v_/*F*_m_** | **_HSD_** | ***F*_v_/*F*_m_** | **_HSD_** |
| **11** | 0.77 ± 0.005 | a | 0.768 ± 0.004 | a | 0.765 ± 0.002 | a | 0.75 ± 0.010 | a |
| **19** | 0.766 ± 0.003 | ab | 0.768 ± 0.004 | a | 0.769 ± 0.003 | a | 0.765 ± 0.005 | a |
| **28** | 0.74 ± 0.004 | bc | 0.747 ± 0.003 | c | 0.737 ± 0.004 | bc | 0.738 ± 0.005 | ab |
| **33** | 0.75 ± 0.004 | abc | 0.749 ± 0.004 | bc | 0.741 ± 0.005 | b | 0.750 ± 0.004 | ab |
| **47** | 0.736 ± 0.009 | c | 0.751 ± 0.004 | bc | 0.716 ± 0.006 | d | 0.744 ± 0.007 | ab |
| **55** | 0.764 ± 0.006 | ab | 0.777 ± 0.003 | a | 0.755 ± 0.004 | ab | 0.755 ± 0.007 | a |
| **61** | 0.747 ± 0.008 | abc | 0.764 ± 0.003 | ab | 0.72 ± 0.006 | cd | 0.721 ± 0.012 | b |

Data are mean ±Standard Error (n=24).

Different letters indicate significant differences at p<0.05.

**Table S7.** Tukey HSD (_HSD_) *post-hoc* test for the interaction effect between genotype, treatment and day for the performance index (PI). Different lowercase letters indicate significant differences between days for each treatment and uppercase letters differences between treatments on the same day (p<0.05) for each genotype.

| **Genotype** | **Day** | **C** | | | **D** | | | **S** | | | **S+D** | | |
| --- | --- | --- | --- | --- | --- | --- | --- | --- | --- | --- | --- | --- | --- |
|  |  | **PI ± SE** | **_HSD-WT_** | **_HSD-BT_** | **PI ± SE** | **_HSD-WT_** | **_HSD-BT_** | **PI ± SE** | **_HSD-WT_** | **_HSD-BT_** | **PI ± SE** | **_HSD-WT_** | **_HSD-BT_** |
| ***M. x giganteus*** | **11** | 1.51 ± 0.167 | abc | A | 1.76 ± 0.167 | ab | A | 1.72 ± 0.081 | a | A | 1.72 ± 0.081 | a | A |
|  | **19** | 2.15 ± 0.145 | a | A | 1.50 ± 0.197 | ab | A | 1.62 ± 0.082 | a | A | 1.62 ± 0.082 | a | A |
|  | **28** | 1.14 ± 0.155 | cd | A | 1.17 ± 0.101 | b | A | 1.08 ± 0.088 | abc | A | 1.08 ± 0.088 | ab | A |
|  | **33** | 1.08 ± 0.124 | cd | A | 1.33 ± 0.157 | ab | A | 1.12 ± 0.086 | ab | A | 1.12 ± 0.086 | abc | A |
|  | **47** | 0.95 ± 0.071 | d | AB | 1.12 ± 0.096 | b | A | 0.58 ± 0.134 | c | B | 0.58 ± 0.13 | abc | AB |
|  | **55** | 1.74 ± 0.051 | ab | A | 1.99 ± 0.182 | a | A | 1.13 ± 0.183 | abc | B | 1.13 ± 0.183 | bc | B |
|  | **61** | 1.21 ± 0.11 | bcd | A | 1.24 ± 0.177 | b | A | 0.88 ± 0.209 | bc | A | 0.88 ± 0.21 | c | A |
| ***M. fluoridulus*** | **11** | 1.38 ± 0.103 | abc | A | 1.14 ± 0.238 | a | A | 1.53 ± 0.154 | a | A | 1.53 ± 0.154 | a | A |
|  | **19** | 1.64 ± 0.123 | ab | A | 1.73 ± 0.326 | a | A | 1.52 ± 0.167 | a | A | 1.52 ± 0.167 | a | A |
|  | **28** | 1.00 ± 0.081 | c | A | 0.85 ± 0.176 | a | A | 0.85 ± 0.119 | ab | A | 0.85 ± 0.119 | ab | A |
|  | **33** | 1.36 ± 0.181 | bc | A | 0.97 ± 0.112 | a | A | 1.05 ± 0.097 | ab | A | 1.05 ± 0.097 | ab | A |
|  | **47** | 1.09 ± 0.087 | bc | A | 0.92 ± 0.190 | a | AB | 0.66 ± 0.129 | b | AB | 0.66 ± 0.129 | b | B |
|  | **55** | 1.98 ± 0.18 | a | A | 1.56 ± 0.267 | a | AB | 1.00 ± 0.183 | ab | BC | 1.01 ± 0.183 | ab | C |
|  | **61** | 1.19 ± 0.147 | bc | A | 0.84 ± 0.185 | a | AB | 0.73 ± 0.184 | b | AB | 0.73 ± 0.184 | b | B |
| ***M. sin. 1*** | **11** | 1.56 ± 0.295 | ab | A | 1.57 ± 0.226 | a | A | 1.51 ± 0.147 | ab | A | 1.51 ± 0.147 | ab | A |
|  | **19** | 1.63 ± 0.206 | ab | A | 1.83 ± 0.078 | a | A | 1.55 ± 0.154 | ab | A | 1.55 ± 0.154 | ab | A |
|  | **28** | 1.14 ± 0.096 | b | A | 1.00 ± 0.097 | b | A | 1.00 ± 0.115 | bc | A | 1.00 ± 0.116 | bc | A |
|  | **33** | 1.21 ± 0.141 | ab | A | 0.99 ± 0.078 | b | A | 1.20 ± 0.170 | abc | A | 1.20 ± 0.17 | abc | A |
|  | **47** | 1.31 ± 0.116 | ab | A | 1.36 ± 0.119 | ab | A | 0.89 ± 0.088 | c | A | 0.89 ± 0.088 | c | A |
|  | **55** | 1.97 ± 0.147 | a | A | 1.94 ± 0.156 | a | A | 1.73 ± 0.156 | a | AB | 1.73 ± 0.156 | a | B |
|  | **61** | 1.33 ± 0.072 | ab | AB | 1.36 ± 0.044 | ab | A | 1.00 ± 0.115 | bc | B | 1.00 ± 0.115 | bc | AB |
| ***M. sin. 2*** | **11** | 2.095 ± 0.238 | ab | A | 2.29 ± 0.363 | a | A | 2.18 ± 0.315 | a | A | 2.18 ± 0.315 | a | A |
|  | **19** | 2.76 ± 0.221 | a | A | 2.39 ± 0.399 | a | A | 2.26 ± 0.238 | a | A | 2.26 ± 0.238 | a | A |
|  | **28** | 1.47 ± 0.196 | bc | A | 1.57 ± 0.240 | ab | A | 1.52 ± 0.196 | abc | A | 1.52 ± 0.197 | abc | A |
|  | **33** | 1.29 ± 0.211 | bc | A | 1.11 ± 0.101 | bc | A | 1.16 ± 0.176 | bc | A | 1.17 ± 0.176 | bc | A |
|  | **47** | 0.97 ± 0.149 | c | A | 0.63 ± 0.082 | c | A | 0.78 ± 0.087 | c | A | 0.78 ± 0.087 | c | A |
|  | **55** | 1.27 ± 0.194 | bc | A | 1.50 ± 0.253 | abc | A | 1.78 ± 0.305 | ab | A | 1.78 ± 0.305 | ab | A |
|  | **61** | 0.83 ± 0.104 | c | A | 0.91 ± 0.192 | bc | A | 1.11 ± 0.138 | bc | A | 1.11 ± 0.138 | bc | A |

Data are mean ±Standard Error (n=6).

Different letters indicate significant differences at p<0.05.

**Table S8.** Tukey HSD (_HSD_) *post-hoc* test for the interaction effect between treatment and genotype for the Malondialdehyde (MDA) content and the Soluble Sugars. Different lowercase letters indicate significant differences between treatments (BT) for each genotype (p<0.05).

| **Genotypes** | **Treatment** | **MDA** | **±SE** | **_HSD_** | **Soluble Sugars** | **±SE** | **_HSD_** |
| --- | --- | --- | --- | --- | --- | --- | --- |
| *M. x giganteus* | C | 0.0125 | 0.0079 | a | 0.1767 | 0.0762 | a |
|  | D | 0.0051 | 0.0016 | a | 0.0817 | 0.0190 | a |
|  | S | 0.0103 | 0.0035 | a | 0.1811 | 0.0445 | a |
|  | S+D | 0.0132 | 0.0092 | a | 0.2806 | 0.2151 | a |
| *M. fluoridulus* | C | 0.0365 | 0.0250 | a | 0.1106 | 0.0244 | a |
|  | D | 0.0060 | 0.0008 | a | 0.0980 | 0.0075 | a |
|  | S | 0.0119 | 0.0047 | a | 0.2099 | 0.0951 | a |
|  | S+D | 0.0062 | 0.0010 | a | 0.1017 | 0.0130 | a |
| *M. sin.* 1 | C | 0.0055 | 0.0023 | a | 0.2187 | 0.0866 | a |
|  | D | 0.0019 | 0.0005 | a | 0.1003 | 0.0217 | a |
|  | S | 0.0028 | 0.0005 | a | 0.0660 | 0.0044 | a |
|  | S+D | 0.0247 | 0.0140 | a | 0.7435 | 0.4562 | a |
| *M. sin.* 2 | C | 0.0276 | 0.0197 | a | 0.2411 | 0.0744 | a |
|  | D | 0.0041 | 0.0008 | a | 0.0703 | 0.0089 | b |
|  | S | 0.0084 | 0.0021 | a | 0.0980 | 0.0198 | ab |
|  | S+D | 0.0089 | 0.0022 | a | 0.1232 | 0.0334 | ab |

Data are mean ±Standard Error (n=6).

Different letters indicate significant differences at p<0.05.
